# Identification and Chemometric Profiling of C_10_- and C_11_-Decalins as Novel Hydrodesulfurization Products for Diesel Spill Environmental Forensics

**DOI:** 10.3390/molecules31173006

**Published:** 2026-08-27

**Authors:** Wei-Hsuan Hsu, Zhi-Han Lin, Steve S.-F. Yu, Shih-Han Lin

**Affiliations:** 1 Institute of Chemistry, Academia Sinica, Taipei 115201, Taiwan; killktv@livemail.tw (W.-H.H.); shorty781008@gmail.com (Z.-H.L.); 2 Department of Applied Chemistry, National Yang Ming Chiao Tung University, Hsinchu 300093, Taiwan; 3 Sustainable Science and Technology (SCST), Taiwan International Graduate Program (TIGP), Academia Sinica, Taipei 115201, Taiwan; 4 Environmental Management Administration, Ministry of Environment, Taipei 100005, Taiwan; shihhan.lin@moenv.gov.tw

**Keywords:** decalins, diagnostic ratios, diesel spills, environmental forensics, hydrodesulfurization (**HDS**), manufacturing era estimation, source identification

## Abstract

This study introduces decahydronaphthalenes (C_10_- and C_11_-decalins; C_0_De and C_1_De) as a novel class of hydrodesulfurization (**HDS**)-associated forensic markers. Diesel-contaminated soil residues from 12 gas station spills and fresh ultra-low-sulfur diesel (**ULSD**) reference samples (2018–2025) in Taiwan were evaluated. The decalin abundances, derived from gas chromatography/mass spectrometry (GC/MS) peak areas, were converted into diagnostic ratios (DRs) using a conserved C_15_ sesquiterpane (BS3) internal reference. These ratios were correlated with conventional fingerprint parameters and alternative **HDS** products to support source identification and manufacturing-era estimation. Notably, Pearson correlations revealed that C_0_De and C_1_De weathering behaviors mimic those of short- (CH-6/CH-7) and medium-chain (CH-9) *n*-alkylcyclohexanes, respectively. Furthermore, C_1_De’s negative correlation with C*_x_*D proxies indicates the premise that rigorous **HDS** processing lowers sulfur concentration while elevating these hydrogenated side products. Expanding on a framework using dimethylbiphenyls (C_2_B) and trimethyltetralins (C_3_T), adding these novel decalin markers in principal component analysis (PCA) maintained manufacturer-specific source discrimination, with collinear loading vectors confirming C_1_De and C_3_T as codependent **HDS** markers. Cross-validated (CV) linear discriminant analysis (LDA) showed that replacing C_3_T with C_0_De or C_1_De improved discrimination among broad sulfur-regulatory manufacturing-era groups, although residual classification uncertainty remained for strongly weathered samples. These results support C_0_De and C_1_De as complementary **HDS**-associated markers for semi-quantitative manufacturing-era assessment rather than exact chronological dating.

## 1. Introduction

Diesel, a middle-distillate petroleum fraction, contains a diverse array of inherent compounds that serve as critical diagnostic markers in environmental forensic investigations [1]. In particular, *n*-alkanes (*n*-C_n_) and isoprenoids, such as phytane (Ph) and pristane (Pr) patterns, selected aromatic compounds or polycyclic aromatic hydrocarbons (PAHs), *n*-alkylcyclohexanes (CH-*n*) and bicyclic sesquiterpanes (BS, sesquiterpenoid hydrocarbons with a decalin-type skeleton and multiple alkyl substituents [2]) are widely used as fuel source and weathering indicators [1,3,4,5,6]. These chemical fingerprints, quantified *via* specific extracted ion mass (EIM) values derived from total ion chromatogram (TIC) full-scan data or acquired directly *via* selected ion monitoring (SIM) mode, are analyzed using gas chromatography/mass spectrometry (GC/MS). The resulting profiles can be utilized for source identification, age dating, or manufacturing era estimation [7,8,9,10,11]. To decouple environmental degradation or evaporation from original fuel characteristics, these profiles are used to construct two distinct classes of diagnostic metrics. **Weathering diagnostic ratios** (**WDR**s) [4,7,12] are formulated by placing a labile, weathering-sensitive compound (such as a linear *n*-alkane) in the numerator against a highly recalcitrant source biomarker in the denominator; this architecture allows investigators to precisely track the preferential loss of light fractions due to evaporation and biodegradation over time. Conversely, **source diagnostic ratios** (**SDR**s) [2,4,5,6,13] consist of mutual ratios between two highly recalcitrant source markers (e.g., isoprenoids like Ph or specific bicyclic sesquiterpanes of BS3 or BS10). Because both components in an **SDR** resist weathering at comparable rates, the ratio remains invariant, thereby preserving the original chemical signature of the source oil. Consequently, through the concurrent utilization of **WDR**s and **SDR**s, investigators can simultaneously evaluate both the environmental weathering state and the source similarity of an unknown diesel spill.

The Christensen–Larsen approach [7], based on weathering diagnostic ratios such as the heptadecane (*n*-C_17_)/Pr ratio, remains one of the most established methods for estimating diesel residence times, providing approximate environmental exposure periods in soil on the scale of a few years. Building on this work, a distinct weathering sequence, based on physical, chemical and biological processes [10,14], was formulated for petroleum products, especially middle distillates such as diesel, kerosene, and No. 2 heating oil, as the Kaplan weathering stages [12,13,15]. This sequential framework characterizes the initial rapid depletion of linear *n*-alkanes and low-molecular-weight PAHs, consistent with traditional depletion profiles and historical residence-time models [16,17,18,19], followed by systematic losses of *n*-alkylcyclohexanes and low-methyl-substituted PAHs. At the advanced stage of environmental weathering (stage 6), microbial degradation extensively consumes highly branched isoprenoids such as Ph and Pr, ultimately leaving only highly resistant biomarkers (e.g., BS, hopanes, and heavy PAHs) essentially intact [2,16,20]. However, existing approaches for age-dating identification, or manufacturing era estimation still rely largely on empirical time–ratio relationships rather than multivariate pattern models [21].

To overcome these constraints, multivariate chemometric models [22,23,24,25,26,27,28,29], particularly principal component analysis (PCA) and linear discriminant analysis (LDA), have been successfully applied to classify petroleum products and distinguish sources using GC-based fingerprints, including two-dimensional gas chromatography (GC×GC) and high-resolution time-of-flight mass spectrometry (HR GC/TOF/MS) [22,23,24,25,29,30]. These tools, alongside state-of-the-art gas chromatography and mass spectrometry techniques, significantly enhance source discrimination, but they have rarely been extended to explicit diesel manufacturing-era scenarios, or incorporated chemical variables that directly reflect hydrotreating and hydrodesulfurization (**HDS**) chemistry [31]. In a recently developed framework from our laboratory [11], PCA was performed on molecular fingerprint profiles by pairing traditional **SDR**s (based on Ph and BS4) with a unique application of refining-specific markers, namely dimethylbiphenyls (C_2_B) and trimethyltetralins (C_3_T). This novel approach segregated diesel samples into discrete clusters corresponding to their respective manufacturers. Furthermore, supervised LDA, incorporating alkyl dibenzothiophenes (C*_x_*D, *x* = 1 and 2) and refining specific markers, such as C_2_B, C_3_T, and trimethylnaphthalenes (C_3_N), enabled classification of samples into inferred regulatory eras spanning the 1993–2011 Sulfur content (*S*-content) control period. However, as global regulatory frameworks shifted entirely to post-2011 modern ultra-low-sulfur regimes (<15 mg *S* kg^−1^ diesel fuel, equivalent to <15 ppm on a mass basis), these specialized aromatic **HDS** markers reached analytical resolution limits under severe hydrotreating conditions; therefore, there is a need to further explore deeper, fully saturated hydrogenation products.

**Scheme 1:**
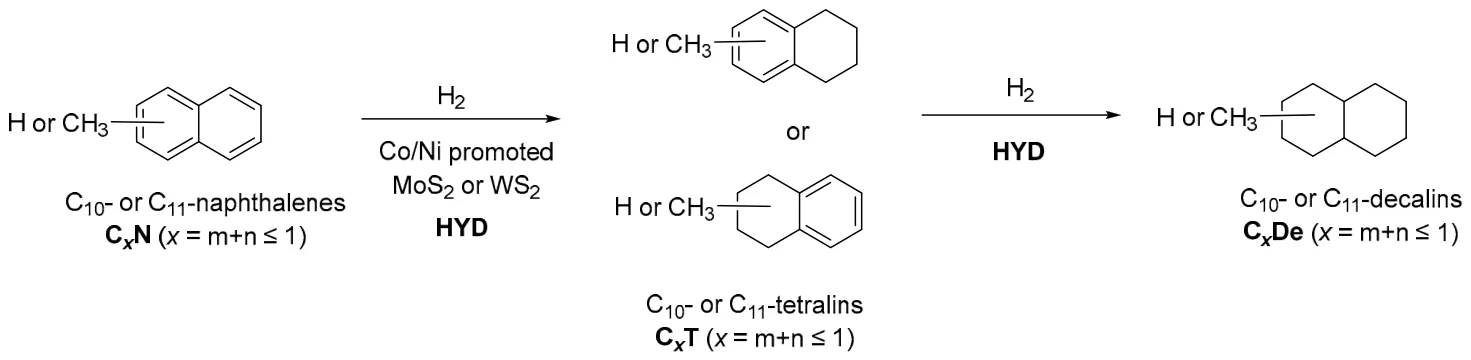
Proposed hydrogenation pathways for the formation of C_10_- and C_11_-decalins (C_0_De and C_1_De) from C_10_- and C_11_-naphthalenes via the corresponding tetralin intermediates under hydrotreating/HDS conditions. The scheme represents literature-supported pathways rather than refinery-specific proprietary processes.

In recent years, increasingly stringent government regulations mandated across the USA, Europe, and China for ultra-low-sulfur diesel (**ULSD**) have driven the global adoption of deep **HDS** over Co/Ni-promoted Mo/W sulfide catalysts [31,32]. For alkyl dibenzothiophenes, direct desulfurization (**DDS**) yields methyl-substituted biphenyls, whereas hydrogenation (**HYD**) routes produce partially saturated intermediates such as cyclohexylbenzenes [31,33,34]; in parallel, the deep hydrogenation of naphthalenes generates tetralins and, under highly severe conditions, fully saturated decahydronaphthalenes (decalins) [35,36,37,38] (Scheme 1). These deeply hydrogenated naphthalenes represent a largely unexplored reservoir of potential post-**HDS** markers in middle-distillate fuels.

Alkyl decahydronaphthalenes (alkyl decalins) are saturated bicyclic naphthalenes that have been detected and structurally characterized in crude oils and related petroleum samples, and have been suggested as potential petroleum biomarkers [14,39]. Hydrogenation and hydrocracking studies [37,40] on fused aromatics show that tetralins, decalins, and methyldecalins can form under conditions relevant to hydrotreating and hydrodesulfurization of diesel-range streams, indicating that C_10_- and short-chain alkyl decalins are plausible, persistent constituents of modern **ULSD** formulations. Furthermore, due to their saturated bicyclic ring structures, similar to BS fingerprints, these alkyl decalins exhibit distinct environmental stability and resistance to microbial cleavage. This resilience can allow them to persist in weathered matrices long after labile linear hydrocarbon fractions have evaporated or degraded, potentially helping to bridge a critical operational gap in historical residence-time models or in manufacturing-era identification.

In this study, the targeted unalkylated and monomethylated decalins (C_10_- and C_11_-decalins, denoted as C_0_De and C_1_De, respectively) were characterized in contaminated soil extracted diesel spills. After validating these compounds against established BS3-normalized **WDR** and **SDR**-based fingerprint markers using Pearson linear correlations, we further applied multivariate statistical tools (PCA and LDA) to an expanded dataset that includes recent ULSD (2018–2025) samples, aiming to improve manufacturer source discrimination and manufacturing era classification. Specifically, the novel deep-hydrogenation markers based on C_10_/C_11_-decalin DRs were evaluated in combination with legacy **HDS**-related markers (C_3_N, C_2_B, C_3_T). This integrated approach evaluates the potential of these composite markers for enhancing the forensic diagnostic resolution of diesel pollution for source identification and linking a spill’s manufacturing date to historical regulatory periods.

## 2. Results and Discussion

### 2.1. Employment of C_10_- and C_11_-Decalins for Forensic Analysis

The analytical framework of this study was extended to evaluate both fresh diesel oils (spanning the 2018–2025 collection period) and weathered diesel spill extracts from the 12 contaminated sites using multivariate statistical analysis for source classification and forensic manufacturing era estimation [11]. This expanded characterization relies on the newly developed diagnostic ratios (DRs) of C_0_De and C_1_De (Table S1 in Supplementary Materials). Chemical markers designated for DR calculations encompassed targeted marker suites including dibenzothiophene (C*_x_*D), biphenyls (C_2_B), indanes (C*_x_*I, *x* = 1 and 2), naphthalenes (C*_x_*N, *x* = 1–4), tetralins (C*_x_*T, *x* = 1–4), and decalins, specifically targeting both unalkylated decalin (C_0_De) and methyl decalins (C_1_De) at the high-abundance EIC of *m*/*z* 95 (corresponding to the [C_7_H_11_]^+∙^ fragment [39,41]), and their respective molecular ion mass-to-charge ratios appeared at the corresponding EICs of *m*/*z* 138 and *m*/*z* 152, respectively (Figure S5, in Supplementary Materials).

Following the calibration of the S-content against high-sulfur diesel feedstock reference standard (6700 ppm), the mean S-contents across the 12 gas stations were determined [11]. Statistical refinements utilizing a Student’s *t*-test allowed for the high-resolution segregation of the contaminated-site data into 19 distinct spill events (Table S5, in Supplementary Materials). Comprehensive operational histories and baseline geographic data regarding these 12 retail stations are detailed in our previous work, and the estimated S-contents (in ppm) for the 12 gas stations were provided in our earlier published results [11].

C_10_- and C_11_-decalins occur naturally in petroleum feedstocks [14,39]. Although refinery-specific catalyst systems and operating conditions are proprietary [31,33,42], these related decalins have been reported to form or become enriched during hydrotreating through the sequential hydrogenation of naphthalene and methylnaphthalenes *via* their corresponding tetralin intermediates (Scheme 1) [36,37,38].

### 2.2. Pairwise Pearson’s Correlation Analysis of r-DRs

**Figure 1:**
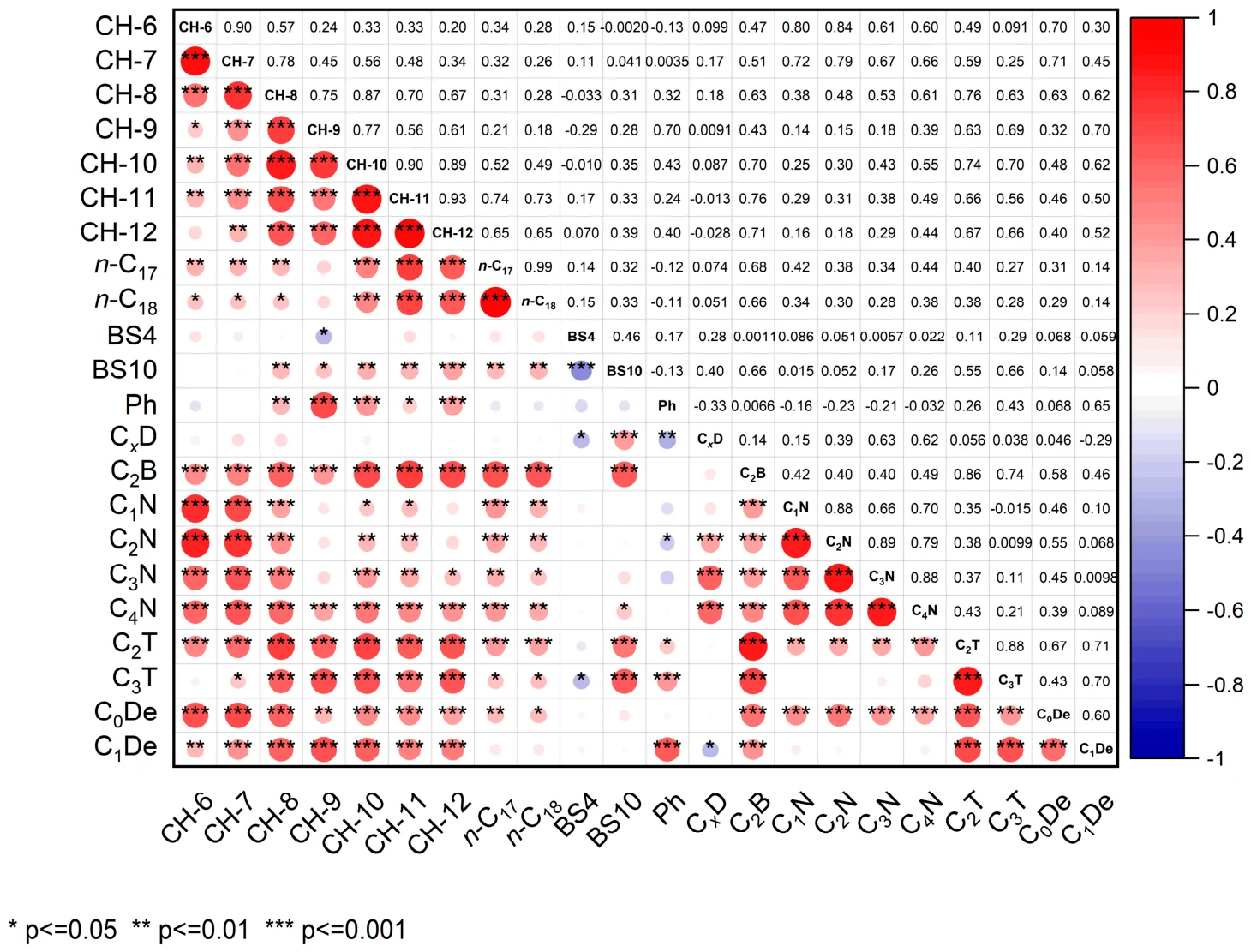
Pairwise correlation matrix of BS3-based relative diagnostic ratios (r-DRs), including *n*-C_17_, *n*-C_18_, BS4, BS10, Ph, C*_x_*D, C_2_B, C_1_N-C_4_N, C_2_T-C_3_T, C_0_De, and C_1_De, derived from 12 sampling sites. The plot illustrates the correlation coefficients (*r*) obtained from linear regression analysis between mutual DRs. Circle size represents the absolute magnitude of Pearson’s *r*-value, whereas color represents the sign and direction of the correlation (red, positive; blue, negative; near-white, weak correlation). Numerical *r* values are displayed in black to improve readability.

Building upon our earlier analysis [11], in which the dataset from the 12 contaminated sites was refined into 19 distinct spill events based on the DR_C*x*D/BS3_ or *S*_RE_ ratios (serving as proxies for *S*-content and regulatory-period classification, respectively), we proceeded with a pairwise correlation analysis based on the C_0_De and C_1_De DRs. Correlation coefficients were systematically calculated across the complete matrix of DRs using BS3 as the conserved internal reference denominator (Figure 1 and Figure S1 in Supplementary Materials).

A series of linear regression analyses were performed to calculate Pearson correlation coefficients (*r*) linking the decalins to various fingerprinting marker *r*-DRs, including *n*-alkanes, isoprenoids, bicyclic sesquiterpanes, *n*-alkylcyclohexanes, PAHs, specifically naphthalenes, and the aromatic **HDS** derivatives (biphenyls and tetralins). The resulting correlation matrix was visualized *via* the dot plots shown in Figure 1, with *r*-values classified into 5 distinct magnitude levels (ranging from 0 to ±1.00) to reveal the mutual correlations among variable *r*-DRs [11,43]. For these comparative evaluations, all DRs were converted to *r*-DRs, where the maximum observed ratio across the entire sample set was defined as the reference baseline value of 1.0. A comprehensive inventory of these DR markers, along with their respective maximum baseline ratios using BS3 as the denominator is provided in Table S1 in Supplementary Materials and the previous work [11].

Importantly, the fresh ULSD reference samples were not included in the Pearson correlation analyses presented in Figure 1 and Figure S1 in Supplementary Materials. These analyses were restricted exclusively to samples collected from the 12 diesel-spill sites in order to evaluate marker relationships within environmentally weathered field residues. The complete field dataset was intentionally retained to evaluate whether the marker relationships remained informative across the continuum of weathering conditions encountered in practical spill investigations, rather than under a single or specific predefined weathering state.

### 2.3. Correlation of Major DR Markers Against the r-DR_C0De/BS3_ and r-DR_C1De/BS3_

To reflect practical forensic conditions, the complete spill-site dataset was retained in the following correlation analyses to assess whether the relationships that C_0_De and C_1_De have with established forensic markers remain informative under the heterogeneous weathering conditions encountered in field investigations.

Linear regression of both *r*-DR_C0De/BS3_ and *r*-DR_C1De/BS3_ against the standard aliphatic proxy *r*-DR*_n_*_-C17/BS3_ revealed correlation coefficients (*r* = 0.31 and 0.14, respectively), indicating weak to negligible correlations (Figure 1, Figures S1 and S2 in Supplementary Materials). Regarding the correlation with isoprenoid Ph, C_0_De exhibits virtually no linear correlation (*r* = 0.067), whereas C_1_De displayed a moderate correlation (*r* = 0.65). Conversely, when correlated against PAHs (C_1_N–C_4_N), *r*-DR_C0De/BS3_ demonstrates low-to-moderate correlations (*r* = 0.39–0.55), while *r*-DR_C1De/BS3_ showed negligible tracking (*r* = 0.011–0.086). These divergent trends suggest that the unalkylated C_0_De marker is more susceptible to weathering than its methylated counterpart, C_1_De, which functions as a relatively stable diagnostic marker akin to isoprenoids. Crucially, both decalin markers exhibit limited correlation to the heavy biomarker BS4. This implies that these unique **HDS** products behave largely independently of the native bicyclic sesquiterpanes background within the diesel matrix.

However, while C_0_De, like C_2_B, C_2_T, and C_3_T, yielded negligible correlation to the regulatory proxy C*_x_*D, C_1_De showed a marginal correlation to weak negative (*r* = –0.29) to *S*-content diagnostic markers (Figure 1 and Figure S1B(2) in Supplementary Materials). This finding fundamentally supports the theoretical negative correlation between **HDS** product indicators and the *S*-content proxy (C*_x_*D), demonstrating that rigorous **HDS** process simultaneously depletes C*_x_*D while elevating specific hydrogenated side products.

Thus, the negligible correlations of C_2_B, C_2_T, C_3_T and C_0_De with the C*_x_*D proxy observed in field applications are primarily attributed to the highly complex degradation variability introduced by profound, long-term environmental weathering processes, which mask the theoretical unweathered baseline. However, because the present correlation analysis intentionally incorporates field samples spanning heterogeneous weathering conditions, these observed correlations should not be interpreted as a direct quantitative measure of **HDS** severity. Likewise, the weak or negligible correlations observed for several **HDS**-related markers may reflect the combined effects of environmental weathering, site-specific matrix conditions, original fuel-composition variability, and refinery history, rather than weathering alone. Importantly, the purpose of the pooled field analysis was to determine whether diagnostically useful relationships persist despite these realistic sources of variability. Within this heterogeneous field dataset, C_3_T and C_1_De nevertheless exhibited a relatively strong correlation (*r* = 0.70), indicating that these two markers retain related compositional information across the investigated spill samples.

Interestingly, C_0_De displayed strong linear correlations with short-chain *n*-alkylcyclohexanes (CH-6 and CH-7), yielding correlation coefficients of 0.70 and 0.71, respectively, representing the lower-end feature of *n*-alkylcyclohexanes [3] (Figure 1 and Figure S1A(1) in Supplementary Materials). Moreover, the *r*-values of CH-6 and CH-7 corresponding to *r*-DRs of *n*-C_17_ and *n*-C_18_ were in a consistent range of 0.26–0.34 to C_0_De (*r* = 0.31). In contrast, C_1_De exhibited moderate to strong correlations with CH-8 (*r* = 0.62) and CH-9 (*r* = 0.70), while the remaining *n*-alkylcyclohexanes demonstrated only weak-to-moderate correlations (Figure 1 and Figure S1B(1) in Supplementary Materials). Notably, linear correlations (*r*) of CH-9 to *n*-C_17_ and *n*-C_18_ were 0.21 and 0.18, respectively, indicating borderline negligible correlations that were comparable to those of C_1_De (*r* = 0.14). The trends align with those revealed for medium-chain *n*-alkylcyclohexanes under deep underground anaerobic conditions [3], and presumably arise because C_10_- and C_11_-decalins are structurally related to cyclohexyl derivatives. A comparative analysis with the *n*-alkylcyclohexane suite implies that decalins share broadly analogous weathering behavior. The distribution experiences progressive preferential losses of normal alkylcyclohexanes from its high end [44] and enhanced levels at the low end [45]. Taken together, these correlation patterns suggest that C_0_De and C_1_De may be relatively less susceptible to environmental weathering than heavier alicyclic compounds such as CH-11 and CH-12 within the investigated field samples.

When benchmarked against other critical refinery **HDS** indicators, C_2_B (derived from the C*_x_*D **HDS** process) exhibited a moderate correlation with C_0_De (*r* = 0.58), and a weaker correlation with C_1_De (*r* = 0.46) (Figure 1, Figures S1A(2) and S1B(2) in Supplementary Materials). For the **HDS** reaction intermediates directly derived from naphthalenes, the alkyltetralins (C_1_T–C_3_T) exhibited a broad correlation range with C_0_De spanning from strong to weak (0.77–0.44), while maintaining consistently strong correlations with C_1_De (*r* = 0.69–0.71) (Figure 1 and Figure S2 in Supplementary Materials). Indanes (C_0_I–C_2_I), which are structural rearrangement products of tetralins [11], exhibited moderate-to-strong linear correlations with C_0_De (*r* = 0.50–0.70) but negligible correlations with C_1_De, similar to C*_x_*N (Figure 1 and Figure S2 in Supplementary Materials). These features suggest that the C_0_De weathering pattern is similar to the depletion profiles of methyl-substituted naphthalenes and indanes, which would be more susceptible to weathering than the other alkylated PAH series [16]. Overall, C_0_De and C_1_De exhibited measurable associations with several established refinery- and **HDS**-related markers, including C_2_B and C_3_T. Together with their persistence across the heterogeneous field samples, these relationships support further evaluation of C_0_De and C_1_De as complementary variables in multivariate forensic models.

### 2.4. Evaluation of C_10_- and C_11_-Decalins for Source Identification via Principal Component Analysis (PCA)

**Figure 2:**
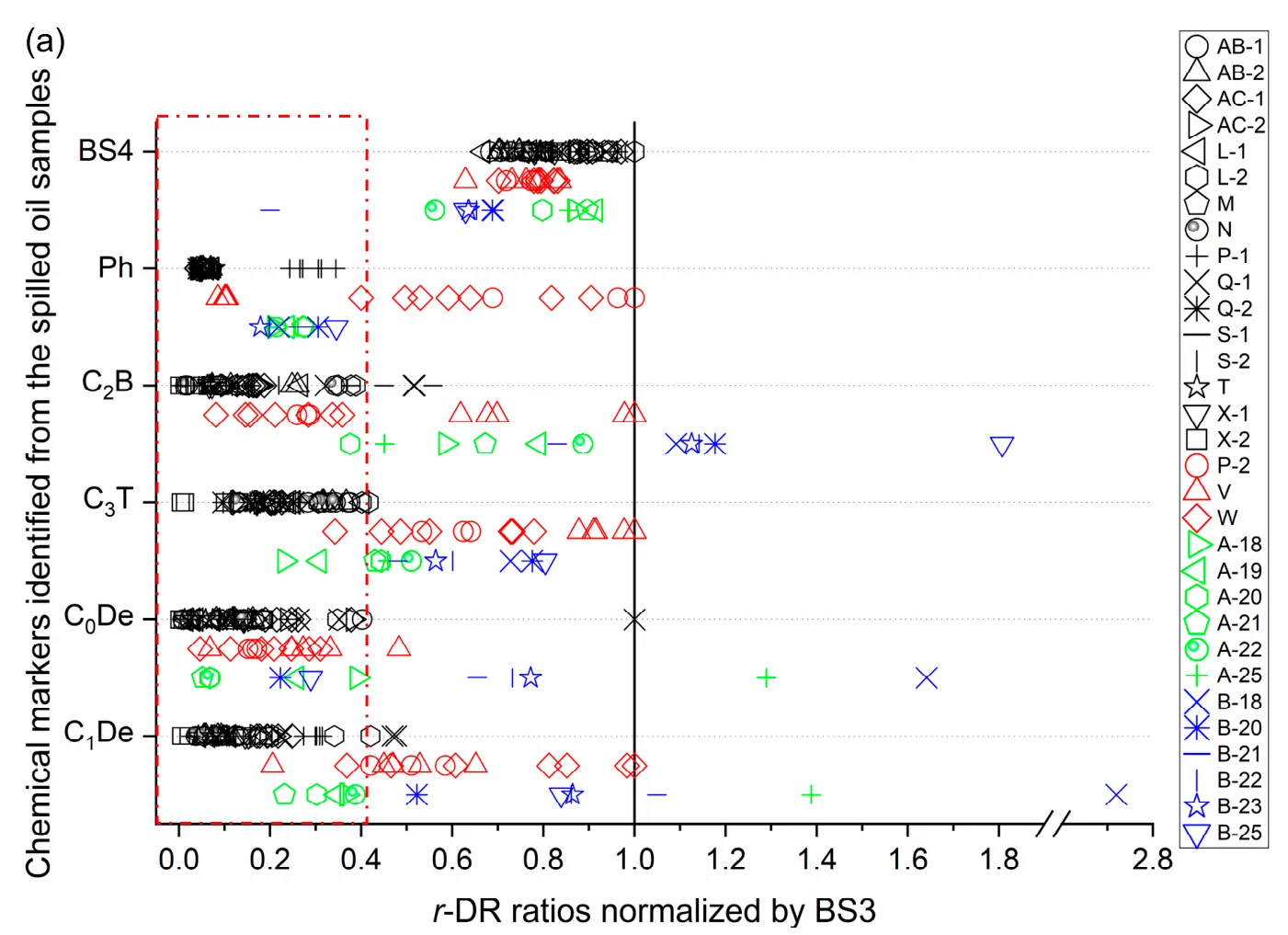

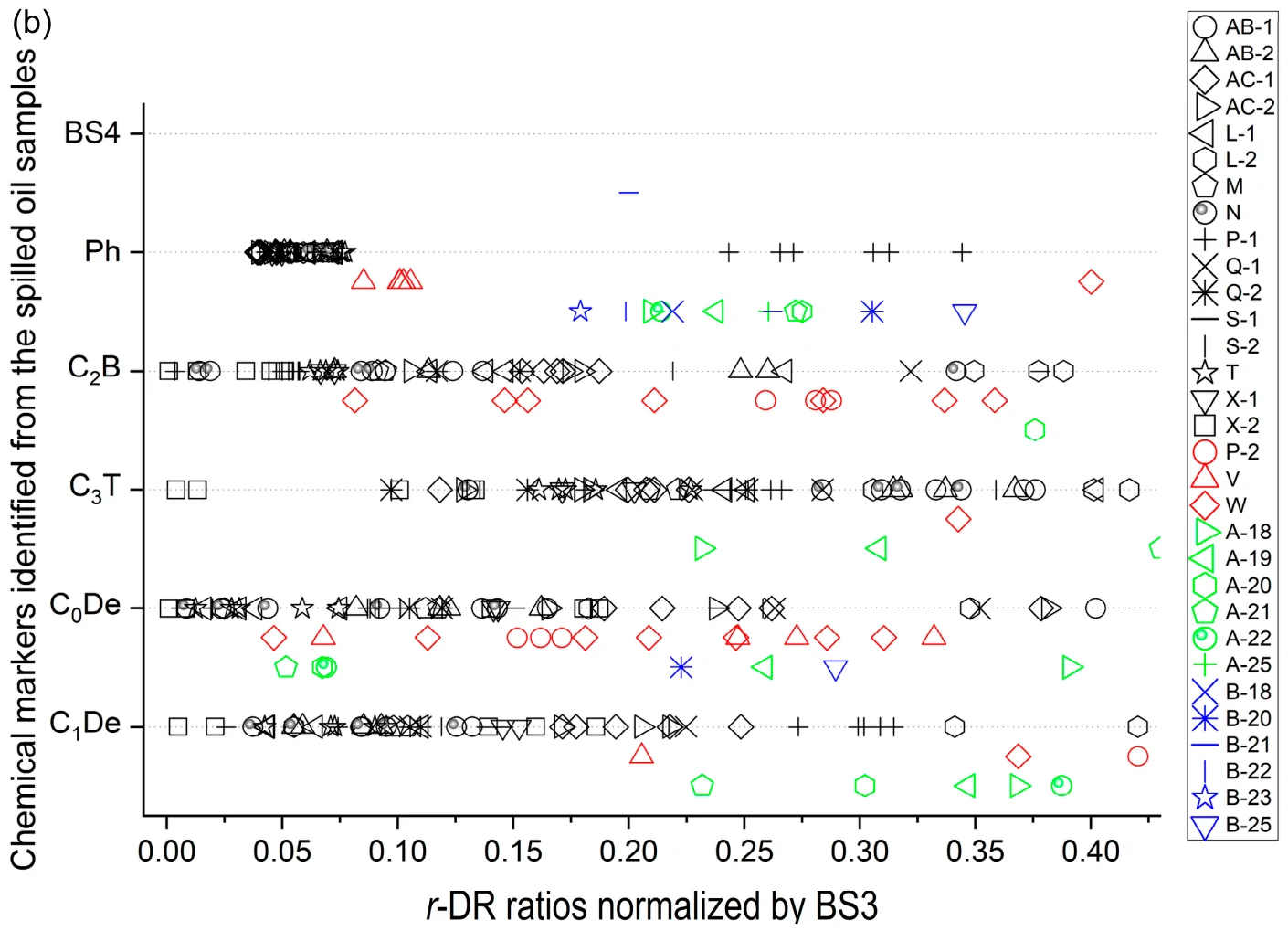
Distribution of BS3-based *r*-DRs, including BS4, Ph, C_2_B, C_3_T, C_0_De, and C_1_De derived from spilled oil (Table S5 in Supplementary Materials) and fresh ULSD (2018–2025) samples attributed to companies A and B. (**a**) Overall distribution across the full *r*-DR range. (**b**) Expanded view of the 0.0–0.4 region (highlighted by the red dashed box in panel (**a**)) to improve visualization of the densely distributed low-ratio observations. **Black** and **red** symbols represent spill-site samples associated with companies **A** and **B**, respectively, whereas **green** and **blue** symbols represent fresh ULSD samples from companies **A** and **B**, labeled by collection year (two-digit numbers).

Previously, appropriate BS3-based DRs were selected to differentiate commercial diesel sources between company **A** and company **B** [11]. Baseline PCA modeling utilizing the *r*-DRs of BS4, Ph, C_2_B, and C_3_T revealed distinct clustering patterns, successfully segregating the fresh reference diesel fuels and the field-weathered soil extracts into the 95% confidence ellipses corresponding to distinct companies **A** and **B**. This baseline configuration attributed the weathered diesel samples from spill sites **V** and **W**, as well as a specific subset of the **P**-site data (designated as Group **P-2**), to company **B**. This clustering was driven by the significantly higher abundances of DR_C2B/BS3_ and DR_C3T/BS3_ characteristic of company **B** formulations with a more rigorous **HDS** process (Figure 2a) [11]. Such manufacturer-associated compositional differences may reflect the combined influence of feedstock characteristics and refinery-specific processing conditions, including **HDS**.

A closely matching trend was observed in the fresh reference diesel samples collected from 2018 to 2025. However, as government sulfur regulations became increasingly stringent over this timeline, shifting chemical profiles altered sample coordinates; for instance, the 2022 fresh reference sample from company **A** (**A-22**) shifted into the 95% confidence ellipse of company **B**, while the 2020 and 2025 company **A** fresh samples (**A-20** and **A-25**) appeared as outliers along the periphery of the company **A** cluster (Figure S3 in Supplementary Materials). Furthermore, the 2025 company **B** fresh diesel sample (**B-25**) also fell completely outside the 95% confidence ellipse of company **B**, shifting towards a higher positive value on PC1 axis.

**Figure 3:**
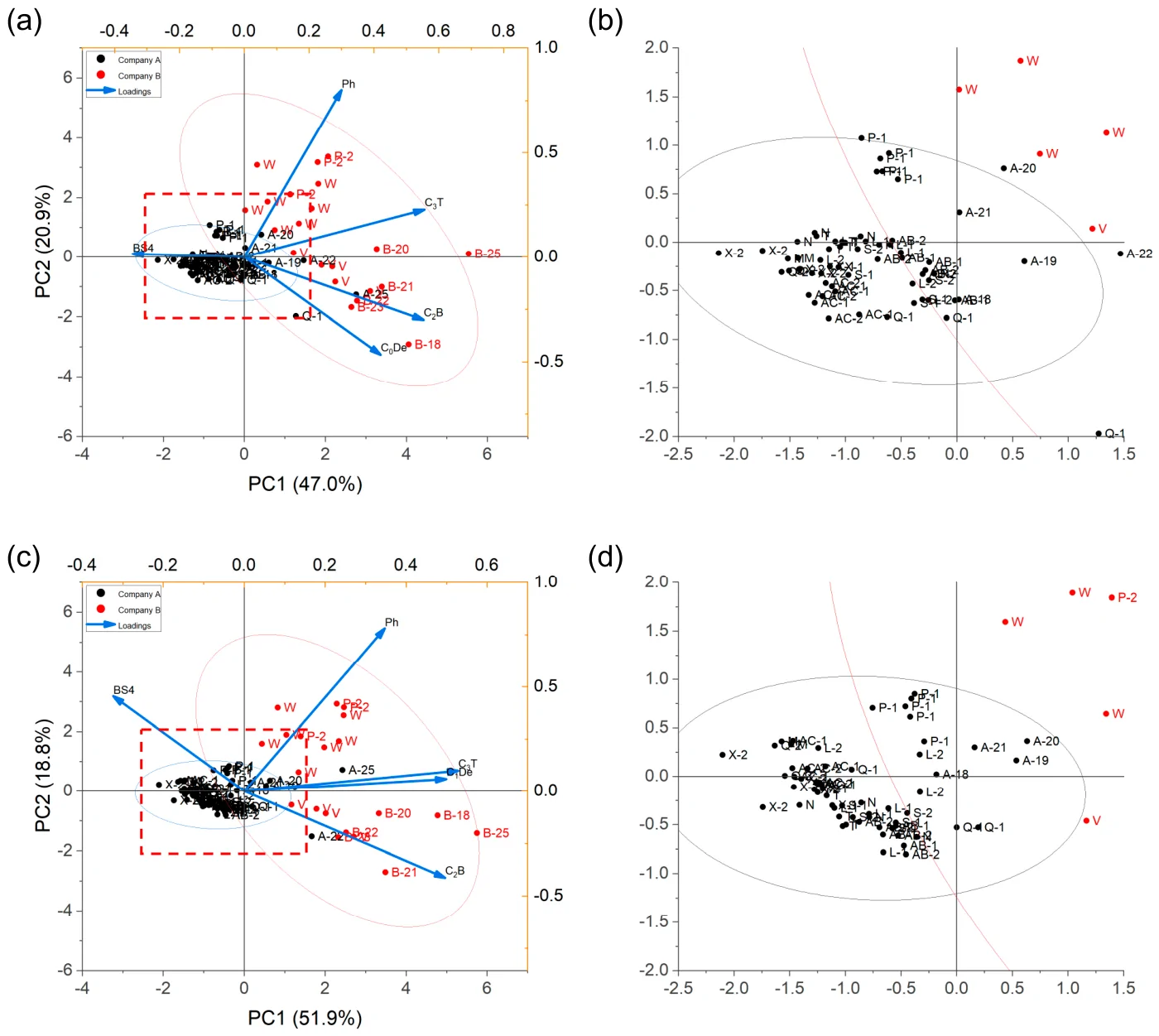
Differentiation of diesel samples (company A vs. company B) using principal component analysis (PCA). The PCA was conducted using five distinct diagnostic ratios as variables: (**a**,**b**) DR_BS4/BS3_, DR_Ph/BS3_, DR_C2B/BS3_, DR_C3T/BS3_, and DR_C0De/BS3_ ratios; (**c**,**d**) DR_BS4/BS3_, DR_Ph/BS3_, DR_C2B/BS3_, DR_C3T/BS3_, and DR_C1De/BS3_ ratios. These were analyzed across 12 spill sites, refined into 19 distinct events (Table S5 in Supplementary Materials), alongside fresh reference diesel samples (denoted by collection year with a two-digit number; e.g., **A-20** represents the company **A** diesel sample collected in 2020). **Black** symbols denote samples associated with company **A**, while **red** symbols denote those associated with company **B**. Ellipses represent the 95% confidence regions of the manufacturer-associated PCA distributions and are used to visualize compositional similarity, group structure, and source-associated positioning of the samples. The red-boxed regions in the complete PCA plots in panels (**a**,**c**) indicate the areas selected for enlarged visualization. Panels (**b**,**d**) show the corresponding zoom-in views of panels (**a**,**c**), respectively, covering PC1 = −2.5 to 1.5 and PC2 = −2 to 2 to facilitate visualization of the densely distributed data points associated primarily with company **A**.

Since diesel-range decalins are predominantly generated *via* sequential hydrogenation of parent naphthalenes during industrial refinery **HDS** processes, we evaluated the diagnostic utility of these alternative markers by systematically adding C_0_De and C_1_De into the PCA framework for comparison. Because the C_0_De DR exhibits a relatively low correlation (*r* = 0.46) with the C_3_T DR, its resulting loading vector projected to the left-hand side of C_2_B, pointing towards the negative axis of PC2. This vector shift caused one low-weathering, high-ratio spill sample from site **Q-1** to migrate away from the company **A** cluster and settle within the 95% confidence ellipse of company **B** (Figure 3a,b). Due to distinct **HDS** selectivity variances between C_3_T and C_0_De, the **A-25** fresh reference diesel sample directly shifted into the company **B**’s ellipse, whereas the **A-20** and **A-22** reference samples positioned themselves along the boundary interface separating the company **A** and company **B** clusters. Meanwhile, the **B-25** fresh diesel sample still fell completely outside the 95% confidence ellipse of company **B**, remaining far away from company **A**’s ellipse.

In contrast, when the C_0_De DR was replaced with the methylated C_1_De marker, its high linear regression correlation (*r* = 0.71) with C_3_T was clearly reflected in the PCA loading plot; the C_1_De vector projected in the same direction as the original C_3_T vector, albeit with a lower magnitude (Figure 3c). Under this configuration, the **A-22** and **A-25** fresh reference oils were redistributed to the center of the company **B**’s 95% confidence ellipse, while the **A-20** fresh oil sample was successfully pulled into the company **A** ellipse (Figure 3c,d). However, this substitution also caused two weathered soil samples originating from company **B** sites **V** and **W**, both with relatively low baseline C_1_De abundances, to fall along the interface or within the confidence ellipse of company **A**, adjacent to company **A**’s fresh diesel samples. Because the baseline DR_C3T/BS3_ abundances for these company **B**-derived **V** and **W** sites are substantially higher than those shown by company **A** (including their fresh diesel samples), co-indexing both C_3_T and C_1_De parameters significantly strengthens the overall source discrimination resolution. Nevertheless, this combination did not counteract the anomalies caused by the extraordinarily high C_1_De levels present in the **A-22** and **A-25** fresh diesels, which still appear within company **B**’s confidence ellipse. Interestingly, the **B-25** fresh diesel sample was located right at the edge of company **B**’s 95% ellipse on the positive side of PC1. These shifts among the PCA data tracks were directly governed by the relative yield and distribution of the C_3_T, C_0_De and C_1_De side products during **LSD** (<500 ppm *S*-content) and **ULSD** (<15 ppm *S*-content) production processes operated by the two companies.

Because company **B** typically utilizes refinery conditions that yield a higher abundance of biphenyls and tetralins, the corresponding decalin abundances tracked at spill sites **V**, **W**, and **P-2** were markedly higher than those recorded at contaminated sites originating from company A (Figure 2). Moreover, the fresh diesel stocks acquired from company **B** under increasingly rigorous HDS modes, necessitated by modern, stringent ULSD environmental standards, corroborate this trend, systematically exhibiting elevated decalin signatures compared to company **A** (Figure 2).

Overall, in the C_0_De-containing model (Figure 3a,b), **A-25** shifted into the company **B** confidence region, whereas **A-20** and **A-22** were located near the boundary between the two groups. In the C_1_De model (Figure 3c,d), **A-22** and **A-25** were positioned within the company **B** confidence region, while **A-20** remained within the company **A** region, indicating partial compositional overlap between the two manufacturers for some recent **ULSD** samples. Notably, incorporation of either C_0_De or C_1_De did not substantially alter the general manufacturer-associated clustering observed in the baseline PCA. Although the 2022 and 2025 fresh ULSD samples from company **A** showed shifts toward or into the company B-associated region, the weathered soil extracts from sites **V**, **W**, and **P-2** consistently remained within the company **B**-associated region across the evaluated PCA marker configurations. This reproducible positioning relative to the manufacturer-associated reference regions provides consistent multivariate evidence for source attribution. Accordingly, within the investigated dataset, the combined and reproducible PCA evidence confirms the attribution of sites **V**, **W**, and **P-2** to company **B**.

To independently quantify the robustness of this manufacturer-discrimination framework and the incremental contribution of the decalin markers, the corresponding predictor sets were further assessed using cross-validated two-class LDA between company **A** and company **B** (Table 1, Tables S6 and S7 in Supplementary Materials). The baseline DR_C2B/BS3_/DR_C3T/BS3_/DR_BS4/BS3_/DR_Ph/BS3_ model yielded a CV accuracy of 95.24% and a balanced accuracy of 96.83%. 4 of the 84 observations were misclassified. The addition of DR_C0De/BS3_ increased these values to 97.62% and 98.41%, reducing the number of misclassified observations from 4 to 2. The addition of DR_C1De/BS3_ yielded a CV accuracy of 96.43% and a balanced accuracy of 97.62%, with 3 misclassified observations. In contrast, simultaneous inclusion of both decalin markers reduced CV accuracy and balanced accuracy to 92.86% and 90.48%, respectively. These results indicate that C_0_De and C_1_De provide incremental manufacturer-discrimination information when incorporated individually, but their combined contribution is not additive.

As C_10_- and C_11_-decalins occur naturally in petroleum feedstocks, the high-sulfur middle-distillate reference with a known total *S*-content of 6700 ppm exhibited DR_C0De/BS3_ and DR_C1De/BS3_ values of 0.14 and 1.04, respectively (Table S1 in Supplementary Materials). These values were substantially lower than the corresponding maximum DR values observed in the investigated dataset (0.78 for C_0_De and 4.91 for C_1_De), indicating that the pre-existing decalin contribution from the feedstock was relatively limited. Therefore, although a feedstock contribution cannot be completely excluded, it is unlikely to dominate the manufacturer-associated compositional differences observed in the PCA models. These datasets were consistent with the LOD/LOQ results, indicating that the quantification limit (S/N ≥ 10) was not attainable for C_1_De-3 to C_1_De-6 (Figure S5 in Supplementary Materials). The other peaks remained detectable at dilution factors ranging from 1/2 to 1/8. Ultimately, the CV two-class LDA data indicated that including the **HDS** markers of C_2_B, C_3_T, C_0_De, and C_1_De can significantly improve the CV accuracy and balanced accuracy to over 90% (Table 1). The results indicated that including the **HDS** markers of the baseline BS3-normalized C_0_De or C_1_De ratios exhibits substantial contributions to the source discrimination between companies **A** and **B**.

### 2.5. Forensic Manufacturing Era Estimation of Spills, Including C_0_De or C_1_De, via Linear Discriminant Analysis (LDA)

To evaluate broad diesel manufacturing era groups aligned with historical sulfur regulations, a baseline supervised LDA was established using the chemical *S*-proxy DR_C*x*D/BS3_, together with the legacy DR_C2B/BS3_, DR_C3T/BS3_ and DR_C3N/BS3_ ratios [11]. This baseline predictive model classified weathered soil extracts from sites **L-1** and **AB-1** as originating from the post-1993 regulatory period, corresponding to a proposed historical *S* concentration range of 1500–3000 ppm. The baseline model subsequently classified spill extracts from sites **AB-2**, **AC-1**, **Q-1**, and **S-1** as post-1997 releases, aligning with the presumably historical 500–1500 ppm *S* regulatory tier. Similarly, the estimated *S*-content profiles for the **AC-2**, **Q-2**, and **S-2** sample groups fall within the 350–500 ppm *S* range, presumably dating these specific manufacturing events to the 1997–1998 regulatory window.

However, definitively distinguishing between the more modern low-*S* limits (10 and 350 ppm) previously was challenging due to low signal-to-noise ratios (<10) for these target markers. Consequently, isolating and resolving individual spill samples from sites **M**, **N**, **W**, **X** (comprising Groups **X-1** and **X-2** (<50 ppm)), and **P** (comprising Groups **P-1** (<50 ppm) and **P-2**), remained problematic in early models (Table S5 in Supplementary Materials) [11].

Cross-validation provided a quantitative assessment of manufacturing-era classification performance. The baseline DR_C2B/BS3_/DR_C3N/BS3_/DR_C3T/BS3_/DR_C*x*D/BS3_ model yielded a CV accuracy of 83.33% and a balanced accuracy of 89.60%. Replacing C_3_T with C_0_De increased these values to 90.48% and 91.54%, respectively, whereas replacement with C_1_De yielded 89.29% and 92.69%. These results indicate improved discrimination of broad sulfur-regulatory groups after substitution with the decalin markers, while also demonstrating that classification remains imperfect (Table S8 in Supplementary Materials).

**Figure 4:**
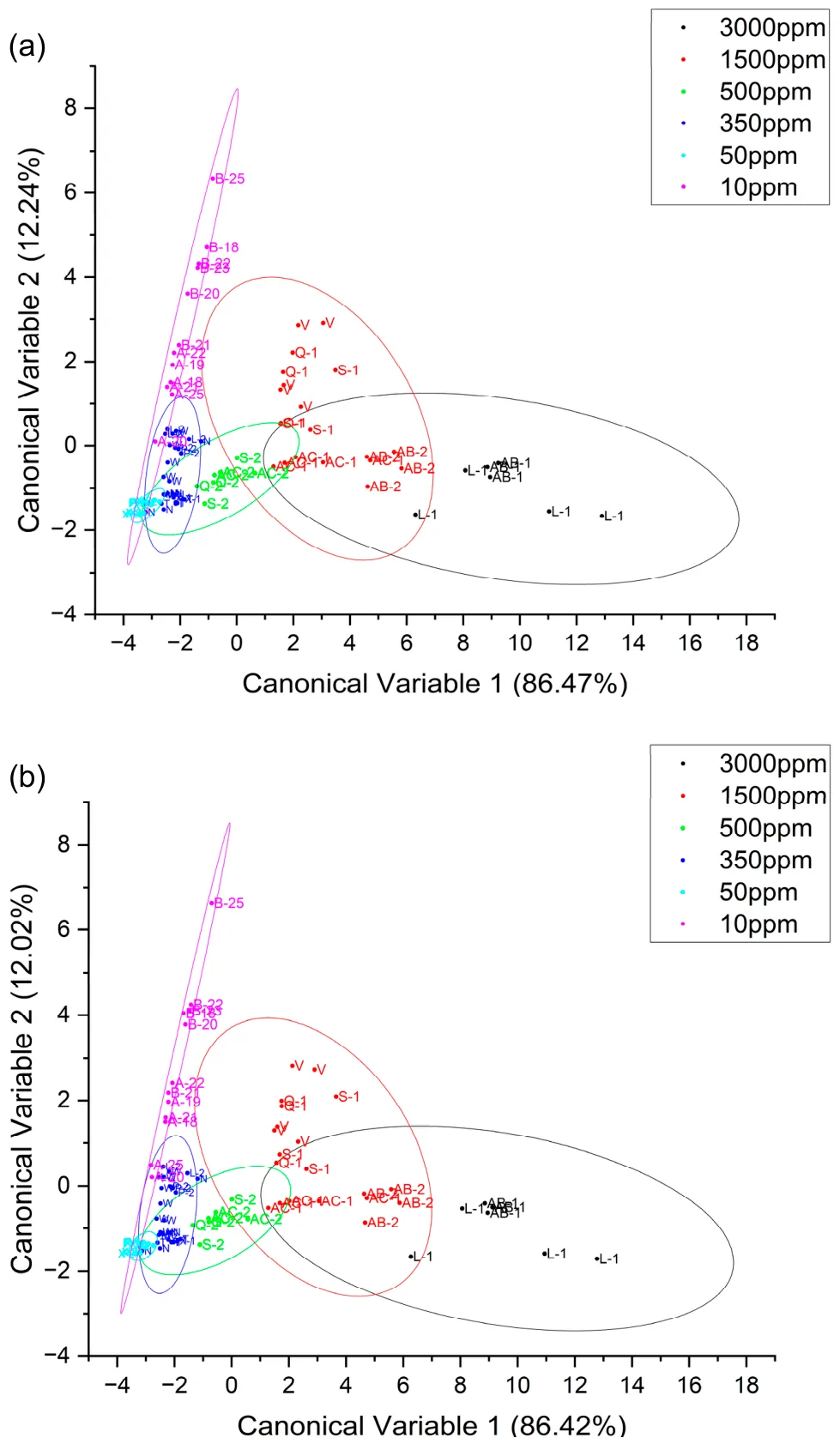
Manufacturing era classification of spilled and fresh diesel samples using linear discriminant analysis (LDA). The LDA model classified samples into groups defined by inferred *S*-content levels corresponding to Taiwan’s historical regulatory period [11]. Four key diagnostic ratios, (**a**) DR_C*x*D/BS3_, DR_C2B/BS3_, DR_C3N/BS3_, and DR_C0De/BS3_; (**b**) DR_C*x*D/BS3_, DR_C2B/BS3_, DR_C3N/BS3_, and DR_C1De/BS3_, were employed as discriminating variables. Marker colors distinguish the different *S*-content regulation zones (e.g., 3000, 1500, 500, 350, 50 and 10 ppm). Ellipses represent the 95% confidence regions of the regulatory groups and illustrate both the overall separation and residual overlap among adjacent manufacturing-era classes.

By systematically substituting the conventional C_3_T DR with the novel C_0_De and C_1_De decalin markers, these ambiguous sites were more clearly segregated into their corresponding supervised *S*-content regulatory regions (Figure 4) [11]. In the earlier LDA configuration utilizing C_3_T, a single sample point from the **Q** site, whose manufacturing era was attributed to the 350–500 ppm *S*-limit regime, anomalously clustered inside the 95% confidence ellipse of the <350 ppm *S* regime. Under the revised decalin-modified model, this point shifted toward the boundary of the 350–500 ppm *S* domain.

The low-sulfur regulatory groups showed improved separation in the decalin-containing LDA models; however, residual overlap and classification uncertainty remained. In particular, the highly weathered **P-1** and **X-2** samples were positioned predominantly within the <50 ppm *S* region, but these assignments should be interpreted cautiously. Both sample groups exhibit relatively low abundances of several HDS-related diagnostic markers, and prolonged environmental weathering may alter their relative marker distributions and shift their positions within the discriminant space. Accordingly, the LDA results for **P-1** and **X-2** are more appropriately interpreted as being broadly consistent with a low-sulfur manufacturing era rather than as precise chronological assignments.

Crucially, all fresh commercial diesel reference points fell tightly within the 95% confidence ellipse of the modern 10 ppm *S* regulatory tier, demonstrating complete separation from the historical 50 ppm and 350 ppm *S* clusters. Because company **B** historically operates under severe refinery parameters that yield higher relative baseline abundances of the **HDS** side-products C_3_T, C_0_De and C_1_De (Figure 2), its fresh diesel formulations occupy a distinct territory. This rigorous **HDS** fingerprint, the 10 ppm *S*-limit 95% confidence ellipse, separates company **B**’s fresh diesel references into the upper region along the second canonical variable axis (Y-axis) in the C_0_De-modified LDA plot (Figure 4a), in sharp contrast to company **A**’s fresh samples, which are positioned in the lower portion of the axis.

Substituting C_3_T with either of the novel decalin DRs, C_0_De or C_1_De consistently maintained the classification of the contaminated soil extracts from site **V** within the 500–1500 ppm *S* regime. However, due to the low baseline abundances of BS3 and BS4 recorded in the site **V** core samples, the true initial *S*-content at this location was refined to below 500 ppm [11]. This adjustment suggests the spilled oils from site **V** were likely manufactured in the 1998–2002 historical window. Historically, the spills at the site **V** originated from a transitioning independent retail gas station that entered the market since 1990, which had not sourced fuel from Taiwan’s major diesel supplier (e.g., company **A**), based on the records [46]. These samples exhibited unique high-abundance normal alkanes (such as *n*-C_17_ and *n*-C_18_ with the baseline *r*-DR = 1.0), whereas the corresponding ratios in fresh modern **ULSD** diesels typically fall below 0.5 [11]. We therefore concluded that the spilled oils from site **V** reflect a specific, unweathered refining formulation with inherently low BS3 levels, a profile that is highly consistent with company **B**’s initial retail market entry.

Additionally, the updated LDA model indicated that the contamination events at the **P-2** site involved diesel whose manufacturing period fell in the 2002–2005 regulatory regime, matching the estimated *S*-content threshold of 350 ppm. These findings further strengthen the source discrimination model for the **P-1** site. This specific site was previously identified *via* PCA as a highly weathered company **A** oil fraction and represents a contamination event established during the historical transition of station ownership from company **A** to company **B**. Due to the lower baseline yields of the characteristic **HDS** products C_3_T, C_0_De and C_1_De inherent to older company **A** processing modes, the spilled diesel matrix at site **P-1** possessed lower structural resistance to environmental weathering. The strongly weathered character of **P-1**, together with its relatively low **HDS**-marker abundances, may have contributed to displacement of these samples within the LDA space. Consequently, the apparent assignment of **P-1** to the low-sulfur regulatory region should be regarded as semi-quantitative and should not be interpreted as an exact manufacturing date.

## 3. Materials and Methods

### 3.1. General Analytical Conditions

The experimental protocols for soil sampling, extraction of fresh and spilled diesel oils, sample preparation, and subsequent GC/MS analysis followed methodologies previously established and validated in our earlier publication [11]. Briefly, 2–5 sampling points were established at each contaminated gas station, with boreholes generally extending to depths of 5–10 m. Continuous soil cores were collected from approximately 1 m below ground surface to the groundwater table, and the intervals showing the highest contamination based on field screening were selected, sealed, stored at approximately 4 °C, and transported to the laboratory. For extraction, approximately 10 g of soil was sequentially extracted with DCM (3 × 10 mL) by ultrasonication for 15 min per cycle. The combined extracts were concentrated to approximately 4 mL by rotary evaporation at 40 °C and subsequently to 1.0 mL under nitrogen. Fresh diesel reference samples (0.50 mL) were separately suspended in 50 mL of an aqueous solution and extracted using DCM (3 × 3 mL), with vortex agitation for 15 min per extraction. The organic extracts were subsequently concentrated to 1.0 mL under nitrogen prior to GC/MS analysis.

Chromatographic separation and mass spectral analysis were performed using gas chromatography/mass spectrometry (GC/MS) systems (Models 6890 GC/5973 MSD, 7890A GC/5975C MSD or 7890B GC/5977B MSD; Agilent Technologies, Santa Clara, CA, USA). Separation was achieved on a DB-1 MS capillary column (60 m × 0.25 mm i.d. × 0.25 μm film thickness). Helium (99.9995%) was used as a carrier gas at a constant flow rate of 1.0 mL min^−1^. The GC oven temperature program was initiated at 40 °C (held for 5 min), ramped at 4 °C/min to a final temperature of 300 °C, where it was maintained isothermally at 300 °C for 10 min. GC/MS data acquisition was performed in full scan mode (scan range: *m*/*z* 50–650). For chemical fingerprinting and diagnostic ratio calculations, characteristic extracted ion mass (EIM, *m*/*z*) profiles for each target analyte were systematically extracted from the total ion chromatogram (TIC) dataset for peak integration. The specific EIMs from the extracted ion chromatograms (EICs) utilized for integration and the corresponding retention times for C_0_De and C_1_De are detailed in the electronic Supplementary Information (Supplementary Materials; Table S1 and Figure S5). All data processing and statistical analyses were performed using OriginPro 2024 (OriginLab Corp., Northampton, MA, USA).

### 3.2. Chemometric Modeling and Diagnostic Calculations [10,11]

A molecular proxy based on alkyl dibenzothiophenes (C*_x_*D: C_1_D and C_2_D) was utilized to infer the *S*-content of the diesel samples on a mass basis. The BS3-normalized alkyl-dibenzothiophene ratio (DR_C*x*D/BS3_) was calibrated against a crude middle-distillate feedstock originating from the Middle East with a known total sulfur content of 6700 mg *S* kg^−1^ fuel (6700 ppm, *w*/*w*) [11]. Assuming proportionality between DR_C*x*D/BS3_ and sulfur content, the inferred sulfur content of each spill sample was estimated relative to the DR_C*x*D/BS3_ value of this reference [11]. This approach represents a reference-based single-point calibration rather than a conventional multi-point calibration curve for absolute sulfur quantification. To mitigate operational errors arising from absolute concentration variances in weathered field spills, target markers were converted into normalized DRs. The highly conserved, biodegradation-resistant C_15_ sesquiterpane derivative (BS3) was chosen as the universal internal reference denominator to compute standard relative indicators (DR_markers_/BS3) [11]. To facilitate cross-site comparison for correlation and regression analysis, each DR (e.g., C_0_De or C_1_De) was normalized against the maximum value observed within the entire dataset to yield the relative diagnostic ratio (*r*-DR) (Table S1, in Supplementary Materials) [11].

### 3.3. Multivariate Statistical Analysis: Principal Component Analysis (PCA) and Linear Discriminant Analysis (LDA)

PCA was conducted to differentiate source attributes between the two dominant fuel suppliers (company **A** vs. **B**). The dataset comprised all soil spill samples from the 12 sites and fresh diesel samples (2018–2025). The PCA model was executed on the correlation matrix computed from the untransformed DRs. The first two principal components (PC1 and PC2) were utilized for the visualization and interpretation of sample clustering.

For manufacturer discrimination, only two predefined classes (company **A** and company **B**) were involved. Because canonical LDA can generate at most *K* − 1 discriminant functions for *K* classes, the two-class problem yields only one non-zero canonical discriminant function and therefore does not provide a natural two-dimensional canonical score plot. PCA was consequently retained for visualization of the manufacturer-associated sample distribution and group overlap on the PC1–PC2 plane, whereas cross-validated LDA was used to quantitatively evaluate classification performance. Thus, PCA and LDA served complementary exploratory and classification purposes, respectively.

To evaluate manufacturing era classification, LDA was performed. The inferred *S*-content level (in ppm (mg *S* kg^−1^)), corresponding to historical government regulatory limits, was defined as the grouping variable [11]. Selected DRs served as predictor variables to generate canonical discriminant functions, enabling evaluation of class separation and misclassification rates over the regulatory timeline. The canonical variables of the diesel samples for LDA were calculated. The derived equations are presented in Table S2 in Supplementary Materials.

### 3.4. Cross-Validation and Evaluation of Classification Performance for Multivariate Statistical Analysis

Because PCA is an unsupervised exploratory method and does not directly provide classification accuracy, the incremental contribution of DR_C0De/BS3_ and DR_C1De/BS3_ to manufacturer discrimination was additionally evaluated using cross-validated two-class LDA between company **A** and **B** [47]. Therefore, four predictor sets corresponding to the PCA source-discrimination framework were compared. These included DR_C2B/BS3_/DR_C3T/BS3_/DR_BS4/BS3_/DR_Ph/BS3_, the baseline set plus DR_C0De/BS3_, the baseline set plus DR_C1De/BS3_, and the baseline set plus both DR_C0De/BS3_ and DR_C1De/BS3_ (Tables S6 and S7 in Supplementary Materials). CV accuracy, class-specific recall, balanced accuracy, and cross-validated confusion counts were used to evaluate classification performance [48,49].

The manufacturing-era LDA models were also evaluated by cross-validation. The original predictor set comprised DR_C2B/BS3_, DR_C3N/BS3,_ DR_C3T/BS3_, and DR_C*x*D/BS3_, whereas the two decalin-containing models replaced C_3_T with C_0_De or C_1_De, respectively. CV accuracy and balanced accuracy were calculated to quantify the discrimination of the broad sulfur-regulatory groups from 10 to 3000 ppm (Table S8 in Supplementary Materials).

For both manufacturer and manufacturing-era classification, performance metrics were calculated from the cross-validated predictions rather than from the re-substitution classifications of the complete training dataset. Leave-one-out cross-validation (LOOCV) was applied, in which each observation was excluded once, the LDA model was fitted using the remaining *N* − 1 observations, and the excluded observation was subsequently assigned to a predicted class [47]. The cross-validated predictions from all iterations were then combined to construct the confusion matrix [48].

Let $n_{kj}$ denote the number of observations belonging to observed class *k* and assigned to predicted class *j*, *N* the total number of observations, and *K* the number of classes. Cross-validated accuracy (CV accuracy) was calculated as the proportion of correctly classified observations:

$$CV\;accuracy\left(\%\right)=\frac{\sum_{k=1}^{K}n_{kk}}{N}\times 100\%.$$

The recall for each class *k* was calculated as

$${Recall}_{k}=\frac{n_{kk}}{\sum_{j=1}^{K}n_{kj}},$$

and balanced accuracy was calculated as the unweighted mean of the class-specific recall values [49]:

$$Balanced\;accuracy\left(\%\right)=\frac{1}{K}\sum_{k=1}^{K}{Recall}_{k}\times 100\%$$

For the two-class manufacturer-discrimination analysis, balanced accuracy therefore corresponds to the mean of the recall values for company **A** and company **B**. For the multiclass manufacturing-era analysis, the same calculation was applied across all sulfur-regulatory groups.

Specifically, for the two-class manufacturer analysis (N=84), CV accuracy and balanced accuracy were calculated as

$$CV\;accuracy\left(\%\right)=\frac{n_{AA}+n_{BB}}{84}\times 100\%,$$

$$Balanced\;accuracy\left(\%\right)=\frac{1}{2}\left[\frac{n_{AA}}{n_{AA}+n_{AB}}+\frac{n_{BB}}{n_{BB}+n_{BA}}\right]\times 100\%,$$

where $n_{AA}$ and $n_{BB}$ represent correctly classified observations from company **A** and company **B**, respectively, whereas $n_{AB}$ and $n_{BA}$ represent the corresponding cross-validated misclassifications.

### 3.5. Quality Assurance, Quality Control, and Analytical Uncertainty

The QA/QC methods were detailed in our previous study [11]. Solvent blank tests ensured that no contamination peaks or carryover occurred during the GC/MS analysis. Furthermore, the evaluation of analytical precision and uncertainty demonstrated that the relative standard deviations (RSDs) remained below 10% across a dynamic sample concentration range of 2.5–20 mg/mL in DCM. For both the raw GC/MS peak area integrations and the calculation of BS3-normalized diagnostic ratios (DRs), the calculated RSDs for most markers, including C_0_De and C_1_De, fell within the 10% uncertainty threshold (Table S3 in Supplementary Materials) [11]. All analytical variations adhered to the 14% repeatability limit prescribed for environmental oil spill forensic analysis [9].

The analytical sensitivity of C_0_De and C_1_De was additionally evaluated using representative fresh **ULSD** and high-sulfur diesel (~6700 ppm *S*) samples. Neat diesel samples were directly diluted with DCM to nominal diesel fractions of 1/2, 1/4, 1/8, 1/16, and 1/32 (*v*/*v*), and analyzed under identical GC/MS conditions. Signal-to-noise (S/N) ratios were determined for C_0_De and six predefined resolved C_1_De peaks. S/N ≥ 3 and S/N ≥ 10 were used as operational criteria for detection and reliable quantification, respectively. To accurately reflect the complex matrix effects, the sensitivity results are reported as matrix-specific, dilution-based operational detection and quantification levels rather than as absolute analyte concentrations derived from neat authentic standards (Table S4 in Supplementary Materials).

## 4. Conclusions

This study establishes unalkylated and monomethylated decahydronaphthalenes (C_0_De and C_1_De) as chemical signatures that improve manufacturing era resolution in environmental forensics for diesel spills, spanning the timeline from legacy **LSD** (<500 ppm) to the modern, post-2011 **ULSD** era. As C_0_De and C_1_De **HDS** markers act as structural analogs to *n*-alkylcyclohexanes, both decalin compounds are less susceptible to deep anaerobic underground weathering than heavier, long-chain *n*-alkylcyclohexanes.

The weak negative association between C_1_De and the C*_x_*D proxy was consistent with an inverse relationship between sulfur-related and hydrogenated markers, although the relative contributions of feedstock composition, refining conditions, and environmental weathering cannot be independently resolved from the present dataset. The baseline PCA framework differentiated broad manufacturer-associated compositional patterns, and the decalin markers provided complementary information when incorporated into the multivariate models.

Several limitations of the present study should be acknowledged. First, the commercial diesel and field-spill samples investigated here were collected primarily within the Taiwanese fuel market, and the transferability of the proposed markers to diesel formulations from other geographic regions remains to be validated. Second, although the field dataset includes highly weathered samples, including samples with strongly depleted conventional weathering indicators, the persistence of C_0_De and C_1_De has not yet been independently verified under controlled extreme-biodegradation conditions. Third, detailed crude-oil feedstock histories and refinery operating parameters were unavailable for the commercial samples; therefore, the independent contributions of crude-oil origin and refining conditions cannot be fully disentangled. Future studies incorporating geographically diverse fuels, known feedstock histories, and controlled biodegradation experiments will be necessary to define the broader applicability of these markers.

Overall, incorporating the decalin markers into the supervised LDA framework improved the discrimination of broad manufacturing-era groups within the investigated dataset. Nevertheless, residual misclassification in some strongly weathered samples indicates that this approach should be regarded as a complementary, semi-quantitative forensic tool rather than a method for exact chronological dating.

## Figures and Tables

**Table 1:** Cross-validated (CV) two-class LDA for evaluating the incremental performance of the C_0_De and C_1_De **HDS** markers in manufacturer discrimination.

Model *^a^*	CV Accuracy (%) *^b^*	Balanced Accuracy (%) *^c^*	Misclassified (n/84) *^d^*
Baseline [11]	95.24	96.83	4
+C_0_De	**97.62**	**98.41**	**2**
+C_1_De	96.43	97.62	3
+C_0_De + C_1_De	92.86	90.48	6

*^a^* The baseline model comprised the BS3-normalized diagnostic ratios of C_2_B, C_3_T, BS4, and Ph (DR_C2B/BS3_, DR_C3T/BS3_, DR_BS4/BS3_, and DR_Ph/BS3_, respectively). The +C_0_De and +C_1_De models were constructed by individually adding DR_C0De/BS3_ or DR_C1De/BS3_ to the baseline predictor set, whereas the “+C_0_De + C_1_De model” included both decalin ratios. *^b^* CV accuracy represents the proportion of correctly classified observations under cross-validation. *^c^* Balanced accuracy was calculated as the mean of the class-specific recall values for company **A** and company **B**. *^d^* “Misclassified” indicates the number of cross-validated predictions that did not match the observed manufacturer among the 84 observations. The highest performance values are shown in bold.

## Data Availability

The data presented in this study are available in the article and Supplementary Materials ^†^. (^†^ Supplementary Information: tables detailing the specific EIM values of C_0_De and C_1_De and their corresponding EIC (extracted ion chromatograms) data; the baseline data used for normalizing the BS3 diagnostic ratios of C_0_De and C_1_De; LOD/LOQ analysis; the estimated *S*-content for the diesel spill samples; cross-validated linear discriminant performance, including the confusion counts for the multivariate statistical analysis; linear correlation plots of the BS3-normalized relative diagnostic ratios based on C_0_De and C_1_De, along with the regression data; and prior PCA and LDA data (excluding C_0_De and C_1_De) used for the classification of spilled and fresh diesel samples according to Taiwan’s historical regulation periods for source identification and manufacturing-era estimation.).

## Supplementary Materials

The following supporting information can be downloaded at https://www.mdpi.com/article/10.3390/molecules31173006/s1. Table S1. Analytical and baseline data for BS3-normalized diagnostic ratios (DRs) of C_0_De and C_1_De. Table S2. Canonical discriminant-function coefficients and explained variance of LDA models using alternative DR_X/BS3_ variables. Table S3. Analytical uncertainty and repeatability of GC/MS peak-area integration, and BS3-normalized diagnostic ratios. Table S4. Dilution-based operational detection and quantification levels of C_0_De and resolved C_1_De peaks in representative diesel matrices. Table S5. The estimated sulfur contents (*S*-contents in ppm) for the diesel spill samples collected from 12 gas stations were derived using the diagnostic ratios DR_CxD/BS3_. This chemical proxy was calibrated against a reference crude middle distillate provided by Taiwan CPC Co., which had an *S*-content of 6700 ppm. Table S6. Cross-validated confusion counts for LDA models used in manufacturer discrimination. Table S7. Sensitivity analysis of cross-validated LDA performance using alternative marker combinations for manufacturer discrimination. Table S8. Cross-validated performance of LDA models for discrimination among sulfur-regulatory manufacturing-era groups. All predictor variables represent BS3-normalized diagnostic ratios, i.e., DR_C2B/BS3_, DR_C3N/BS3_, DR_C3T/BS3_, DR_CxD/BS3_, DR_C0De/BS3_, and DR_C1De/BS3_. A check mark (✓) indicates inclusion of the corresponding diagnostic ratio in the LDA model, whereas an em dash (—) indicates exclusion. The baseline model comprised DR_C2B/BS3_, DR_C3N/BS3_, DR_C3T/BS3_, and DR_CxD/BS3_. In the decalin-substitution models, DR_C3T/BS3_ was replaced by either DR_C0De/BS3_ or DR_C1De/BS3_. CV accuracy represents overall cross-validated classification accuracy, whereas balanced accuracy represents the mean of the class-specific recall values across the sulfur-regulatory groups. The highest performance values are shown in bold. Figure S1. Linear regression analysis of BS3-normalized relative diagnostic ratios using C_0_De and C_1_De as reference variables. The scatter plots illustrate the correlations between individual *r*-DRmarker/BS3 values and the reference *r*-DRs. Solid lines represent the least-squares linear fits, while shaded regions indicate the 95% confidence bands. Pearson linear correlation coefficients (*r*) and slopes are provided in the figure legend. Figure S2. An expanded pairwise Pearson correlation matrix including BS3-normalized C_0_De and C_1_De relative diagnostic ratios (*r*-DRs) alongside the previous established markers for comparison [11]. Asterisks indicate statistical significance levels: *p* ≤ 0.05, *p* ≤ 0.01, and *p* ≤ 0.001. Figure S3. Baseline PCA source identification model using four distinct BS3-normalized DRs. This baseline PCA model used DR_BS4/BS3_, DR_Ph/BS3_, DR_C2B/BS3_, and DR_C3T/BS3_ as variables for the source identification of spilled and fresh diesel samples [11]. This model is provided as the baseline reference for comparison with the decalin-modified PCA models. Figure S4. Baseline LDA manufacturing-era classification model using the conventional HDS DR_X/BS3_ variable. The baseline LDA model uses DR_C*x*D/BS3_, DR_C2B/BS3_, DR_C3N/BS3_, and DR_X/BS3_ as predictor variables, with X set to C_3_T. This model is provided as the baseline reference for comparison with the subsequent LDA models in which the C_3_T DR is substituted with C_0_De or C_1_De DRs [11]. Figure S5. Representative extracted ion chromatograms (EICs) of C_0_De and C_1_De generated from full-scan GC/MS data. DR calculations for C_0_De and C_1_De in source identification and manufacturing-era estimation were based on the EIC of *m*/*z* =95. The additional superimposed EICs at *m/z* 95, 138, and 152 were included as confirmatory traces for the molecular ion masses of C_0_De (*m*/*z* 138) and C1De (*m*/*z* 152), respectively.
