# Supplementary material for: Identification and Chemometric Profiling of C10- and C11-Decalins as Novel Hydrodesulfurization Products for Diesel Spill Environmental Forensics
Source: Molecules. 2026 Aug 27;31(17):3006. doi: 10.3390/molecules31173006 (PMC13567416; doi:10.3390/molecules31173006)

Electronic Supplementary Information

Identification and Chemometric Profiling of C_{10}- and C_{11}-Decalins as Novel Hydrodesulfurization Products for Diesel Spill Environmental Forensics

Wei-Hsuan Hsu[1,2,3], Zhi-Han Lin[1], Steve S.-F. Yu[1,3,*] and Shih-Han Lin[4]

[1]Institute of Chemistry, Academia Sinica, Taipei 115201, Taiwan, R.O.C.
[2]Department of Applied Chemistry, National Yang Ming Chiao Tung University, Hsinchu 300093, Taiwan, R.O.C.
[3]Sustainable Science and Technology (SCST), Taiwan International Graduate Program (TIGP), Academia Sinica, Taipei 115201, Taiwan, R.O.C.
[4]Environmental Management Administration, Ministry of Environment, Taipei 100005, Taiwan, R.O.C.

Keywords: decalins; diagnostic ratios; diesel spills; environmental forensics; hydrodesulfurization (HDS); manufacturing era estimation; source identification

[*]Corresponding author
E-mail address: sfyu@gate.sinica.edu.tw (S. S.-F. Yu)

Tables

Table S1. Analytical and baseline data for BS3-normalized diagnostic ratios (DRs) of C_0De and C_1De.

Markers (spilled site)	**C_0De (Q-1)**	**C_1De (W)**
DR_{max}	0.78	4.91
High-S reference DR (6700 ppm S)	0.14	1.04
t_R (min)	25.48	27.98–29.96
Quantifier EIM (m/z)	95	95

This table lists the key analytical parameters of the chemical markers used for DR calculation (marker/BS3), including the extracted ion mass (EIM, m/z) used for GC/MS peak integration, retention time (t_R, min), and maximum integration peak-area values used as baseline for the r-DR normalization. In comparison, this table also provides the BS3-normalized DRs measured in the high-sulfur middle-distillate reference (6700 ppm S) which was used for sulfur-proxy calibration. Note that these values were not used as the maximum values for r-DR normalization.

Table S2. Canonical discriminant-function coefficients and explained variance of LDA models using alternative $DR_{X/BS3}$ variables.

X	Canonical Variable	$DR_{CxD/BS3}$	$DR_{C2B/BS3}$	$DR_{C3N/BS3}$	$DR_{X/BS3}$	Percentage of Variance (%)
C_3T	1	3.15	0.74	0.038	0.011	82.08
	2	-0.20	2.68	0.036	-0.17	15.88
C_0De	1	3.18	0.48	0.026	0.57	86.47
	2	-0.19	1.56	0.060	0.97	12.24
C_1De	1	3.13	0.52	0.034	-0.015	86.42
	2	-0.23	1.61	0.074	0.015	12.02

The LDA models were constructed using a common predictor set composed of $DR_{CxD/BS3}$, $DR_{C2B/BS3}$, $DR_{C3N/BS3}$, and $DR_{X/BS3}$, where X represents the substitutable **HDS**-related marker (C_3T, C_0De, or C_1De). The coefficients for Canonical Variable 1 and Canonical Variable 2 are listed with the percentage of variance explained by each canonical variable.

Table S3. Analytical uncertainty and repeatability of GC/MS peak-area integration, and BS3-normalized diagnostic ratios.

	Peak area uncertainty (RSD (%))						DR uncertainty (RSD (%))					
	Instrumental Precision			Method Reproducibility			Instrumental Precision			Method Reproducibility		
**samples**[a]	**A***	**B***	**S***	**A***	**B***	**S***	**A***	**B***	**S***	**A***	**B***	**S***
**C_0De**	1.30	4.98	3.53	5.46	1.52	2.00	0.78	3.44	2.56	5.46	1.52	2.00
**C_1De**	0.90	0.96	4.95	5.49	2.35	1.28	1.79	0.74	4.05	5.49	2.35	1.28

[a] **A*** and **B*** denote fresh diesel samples collected from companies A and B, respectively, whereas **S*** denotes the high-sulfur (6700 ppm) diesel reference sample.

Table S4. Dilution-based operational detection and quantification levels of C_0De and resolved C_1De peaks in representative diesel matrices.

	High-S diesel peaks		Fresh ULSD peaks	
Marker	S/N ≥ 3	S/N ≥ 10	S/N≥3	S/N≥10
C_0De	1/16	1/2	1/32	1/32
C_1De-1	1/32	1/8	1/32	1/32
C_1De-2	1/32	1/4	1/32	1/32
C_1De-3	1/2	NR	1/32	1/16
C_1De-4	1/2	NR	1/32	1/16
C_1De-5	1/4	NR	1/32	1/8
C_1De-6	1/8	NR	1/32	1/32

Values represent the lowest tested nominal diesel fraction satisfying the specified S/N criterion. S/N ≥ 3 and S/N ≥ 10 were used as operational criteria for detection and reliable quantification, respectively. A value of 1/32 indicates that the corresponding criterion was still satisfied at the lowest diesel fraction tested; therefore, the actual operational threshold may be lower than 1/32. NR, not reached within the tested dilution range. These values represent matrix-specific, dilution-based operational sensitivity levels rather than absolute concentration-based LOD/LOQ values derived from neat authentic standards.

Table S5. **The estimated sulfur contents (*S*-contents in ppm) for the diesel spill samples collected from 12 gas stations were derived using the diagnostic ratios $DR_{CxD/BS3}$.** This chemical proxy was calibrated against a reference crude middle distillate provided by Taiwan CPC Co., which had an *S*-content of 6700 ppm.

Oil-spilled gas stations	*n*	Mean	Standard Deviation (SD)	*S*-content (ppm) of historic regulation limits
AB-1	3	1963.67	20.07	3,000
AB-2	4	1334.75	83.31	1,500
AC-1	5	903.20	199.08	1,500
AC-2	3	463.00	97.70	500
L-1	4	2120.50	427.13	3,000
L-2	3	93.00	70.83	350
M	2	124.50	11.50	350
N	5	154.60	72.42	350
P-1	6	30.67	2.87	50
P-2	3	136.67	17.15	350
Q-1	3	649.67	25.32	1,500
Q-2	2	339.50	45.50	350
S-1	3	833.33	99.37	1,500
S-2	2	423.00	61.00	500
T	5	192.00	41.39	350
V	5	719.80	68.64	1,500
W	7	96.71	18.77	350
X-1	2	202.00	28.00	350
X-2	5	48.40	31.15	50

a

Table S6. Cross-validated confusion counts for LDA models used in manufacturer discrimination.

Model	A→A	A→B	B→A	B→B
Baseline	59	4	0	21
+ C_0De	61	2	0	21
+ C_1De	60	3	0	21
+ C_0De + C1De	60	3	3	18

Model definitions are identical to those reported in **Table 1**. Briefly, the baseline model comprised $DR_{C2B/BS3}$, $DR_{C3T/BS3}$, $DR_{BS4/BS3}$, and $DR_{Ph/BS3}$; $DR_{C0De/BS3}$ and $DR_{C1De/BS3}$ were then added individually or simultaneously as indicated. All variables therefore represent BS3-normalized diagnostic ratios. Rows represent the observed manufacturer, and columns represent the cross-validated predicted manufacturer; thus, A→B indicates a company **A** observation classified as company **B**, whereas B→A indicates a company **B** observation classified as company **A**.

Table S7. Sensitivity analysis of cross-validated LDA performance using alternative marker combinations for manufacturer discrimination.

Model	No. of DRs[a]	C_2B	C_3T	BS4	Ph	C_0De	C_1De	CV accuracy (%)	Balanced accuracy (%)
1	3	✓	—	✓	✓	—	—	92.86	90.48
2		✓	✓	✓	—	—	—	92.86	90.48
3		✓	✓	—	✓	—	—	94.05	96.03
4		✓	✓	—	—	✓	—	89.29	83.33
5		✓	✓	—	—	—	✓	94.05	91.27
6		✓	—	✓	—	✓	—	91.67	88.10
7		✓	—	✓	—	—	✓	94.05	91.27
8		✓	—	—	✓	✓	—	82.14	72.22
9		✓	—	—	✓	—	✓	91.67	86.51
10		—	✓	✓	✓	—	—	90.48	88.89
11		—	✓	✓	—	✓	—	90.48	88.89
12		—	✓	✓	—	—	✓	96.43	97.62
13		—	✓	—	✓	✓	—	92.86	90.48
14		—	✓	—	✓	—	✓	95.24	93.65
15		—	—	✓	✓	✓	—	91.67	88.10
16		—	—	✓	✓	—	✓	91.67	89.68
17	4	✓	✓	✓	✓	—	—	95.24	96.83
18		✓	—	✓	✓	✓	—	91.67	89.68
19		✓	—	✓	✓	—	✓	91.67	89.68
**20**	**5**	**✓**	**✓**	**✓**	**✓**	**✓**	—	**97.62**	**98.41**
21		✓	✓	✓	✓	—	✓	96.43	97.62
22	6	✓	✓	✓	✓	✓	✓	92.86	90.48

All predictor variables represent BS3-normalized diagnostic ratios, i.e., $DR_{C2B/BS3}$, $DR_{C3T/BS3}$, $DR_{BS4/BS3}$, $DR_{Ph/BS3}$, $DR_{C0De/BS3}$, and $DR_{C1De/BS3}$. [a]No. of DRs indicates the number of BS3-normalized diagnostic ratios included in each LDA model. A check mark (✓) indicates that the corresponding diagnostic ratio was included in the LDA model, whereas an em dash (—) indicates that it was excluded. CV accuracy represents the proportion of correctly classified observations under cross-validation, and balanced accuracy was calculated as the mean of the class-specific recall values for company **A** and company **B**. The four principal models discussed in the main text are shown as Models 17 and 20–22. The highest performance values are shown in bold.

Table S8. Cross-validated performance of LDA models for discrimination among sulfur-regulatory manufacturing-era groups. All predictor variables represent BS3-normalized diagnostic ratios, i.e., $DR_{C2B/BS3}$, $DR_{C3N/BS3}$, $DR_{C3T/BS3}$, $DR_{CxD/BS3}$, $DR_{C0De/BS3}$, and $DR_{C1De/BS3}$. A check mark (✓) indicates inclusion of the corresponding diagnostic ratio in the LDA model, whereas an em dash (—) indicates exclusion. The baseline model comprised $DR_{C2B/BS3}$, $DR_{C3N/BS3}$, $DR_{C3T/BS3}$, and $DR_{CxD/BS3}$. In the decalin-substitution models, $DR_{C3T/BS3}$ was replaced by either $DR_{C0De/BS3}$ or $DR_{C1De/BS3}$. CV accuracy represents overall cross-validated classification accuracy, whereas balanced accuracy represents the mean of the class-specific recall values across the sulfur-regulatory groups. The highest performance values are shown in bold.

Model	C_2B	C_3N	C_3T	C_xD	C_0De	C_1De	CV accuracy (%)	Balanced accuracy (%)
Baseline	✓	✓	✓	✓	—	—	83.33	89.60
$C_3T \rightarrow C_0De$	✓	✓	—	✓	✓	—	**90.48**	91.54
$C_3T \rightarrow C_1De$	✓	✓	—	✓	—	✓	89.29	**92.69**
+ C_0De	✓	✓	✓	✓	✓	—	83.33	89.60
+ C_1De	✓	✓	✓	✓	—	✓	83.33	89.60

Figures

Figure S1. Linear regression analysis of BS3-normalized relative diagnostic ratios using C_0De and C_1De as reference variables. The scatter plots illustrate the correlations between individual *r*-$DR_{marker/BS3}$ values and the reference *r*-DRs. Solid lines represent the least-squares linear fits, while shaded regions indicate the 95% confidence bands. Pearson linear correlation coefficients (*r*) and slopes are provided in the figure legend.

A. C_{10}-decalin (C_0De; *vs.* *r*-$DR_{C0De/BS3}$)

Figure S1A(1). Fingerprint markers: *n*-alkylcyclohexanes (CH-6–CH-12), *n*-alkanes [*n*-heptadecane (*n*-C_{17}) and *n*-octadecane (*n*-C_{18})], bicyclic sesquiterpanes (BS4 and BS10), and the isoprenoid phytane (Ph).

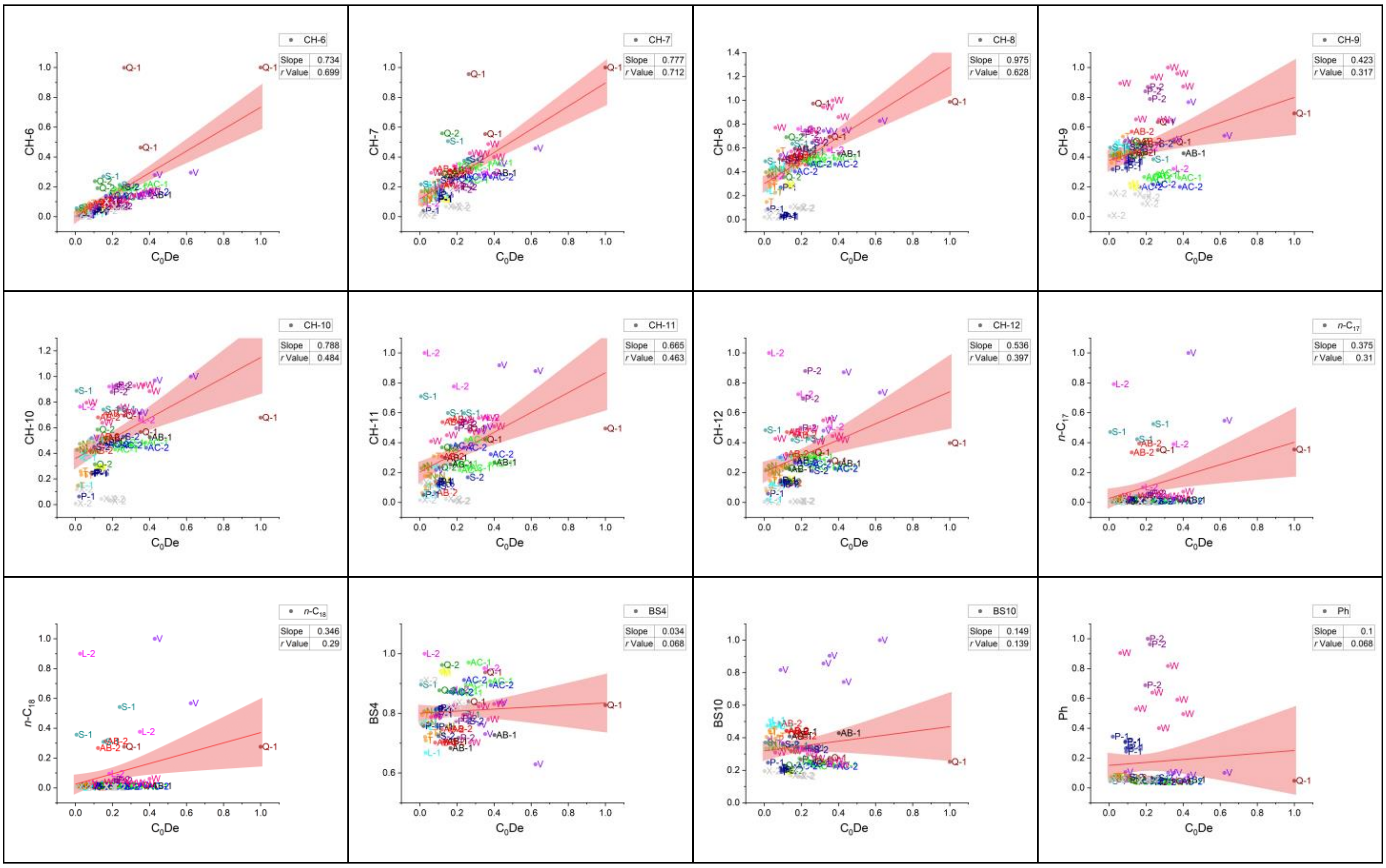

Figure S1A(2). Fingerprint markers: PAHs (C_xD (C_1D and C_2D); C_1N–C_4N) and **HDS** product markers (C_2B, C_2T–C_3T).

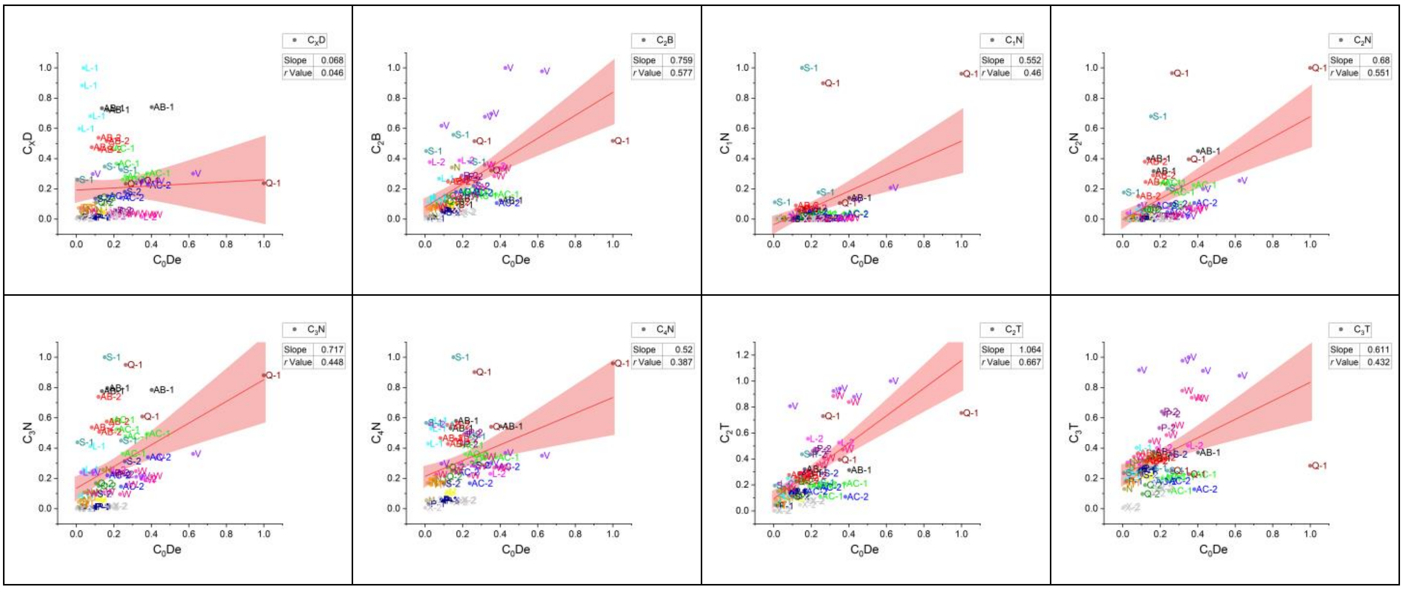

B. C11-decalins (methyldecalins; *vs.* *r*-DR$_{C1De/BS3}$)

Figure S1B(1). Fingerprint markers: *n*-alkylcyclohexanes (CH-6–CH-12), *n*-alkanes [*n*-heptadecane (*n*-C_{17}) and *n*-octadecane (*n*-C_{18})], bicyclic sesquiterpanes (BS4 and BS10), and the isoprenoid phytane (Ph).

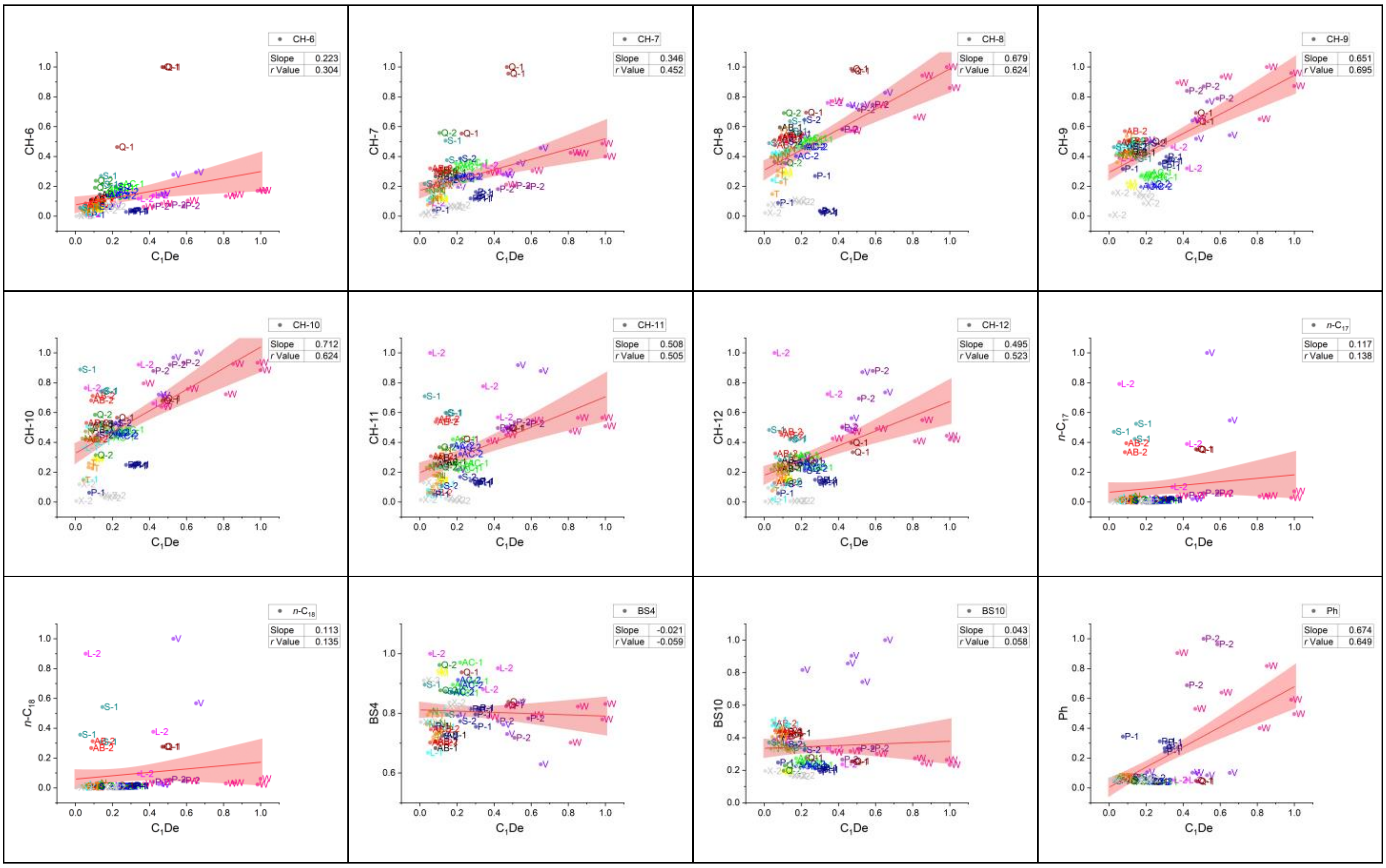

Figure S1B(2). Fingerprint markers: PAHs (C_xD (C_1D and C_2D); C_1N–C_4N) and **HDS** product markers (C_2B, C_2T–C_3T).

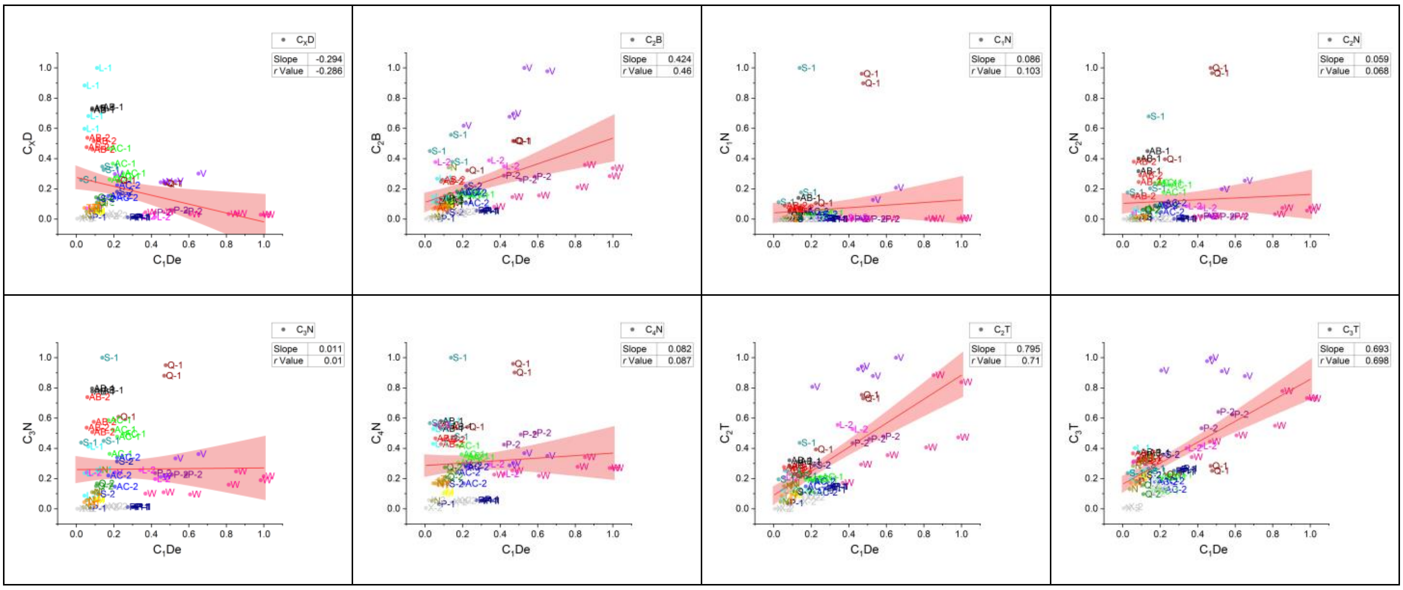

Figure S2. An expanded pairwise Pearson correlation matrix including BS3-normalized C_0De and C_1De relative diagnostic ratios (*r*-DRs) alongside the previous established markers for comparison [11]. Asterisks indicate statistical significance levels: $p \leq 0.05$, $p \leq 0.01$, and $p \leq 0.001$.

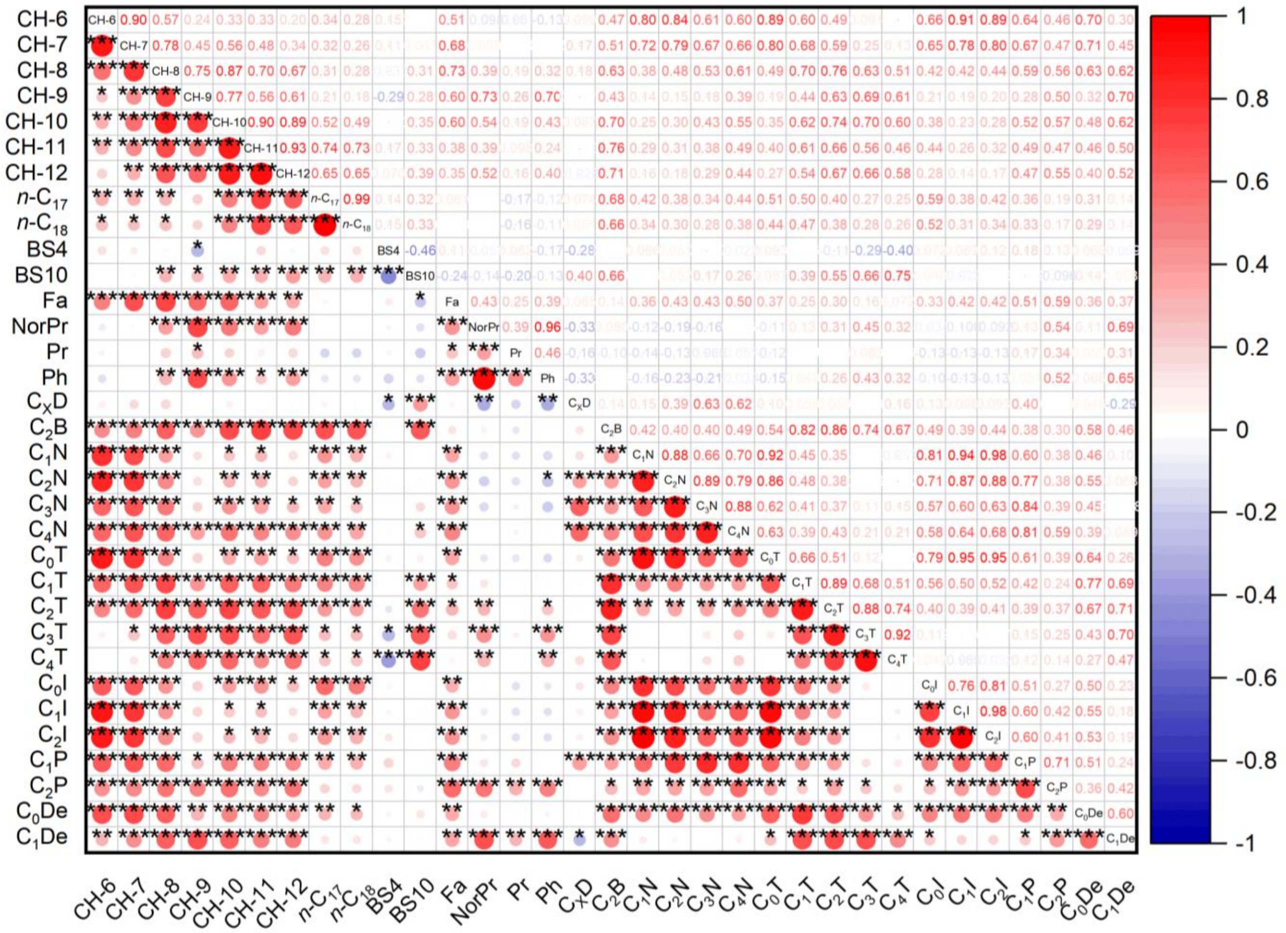

* p<=0.05 ** p<=0.01 *** p<=0.001

Figure S3. Baseline PCA source identification model using four distinct BS3-normalized DRs. This baseline PCA model used $DR_{BS4/BS3}$, $DR_{Ph/BS3}$, $DR_{C2B/BS3}$, and $DR_{C3T/BS3}$ as variables for the source identification of spilled and fresh diesel samples [11]. This model is provided as the baseline reference for comparison with the decalin-modified PCA models.

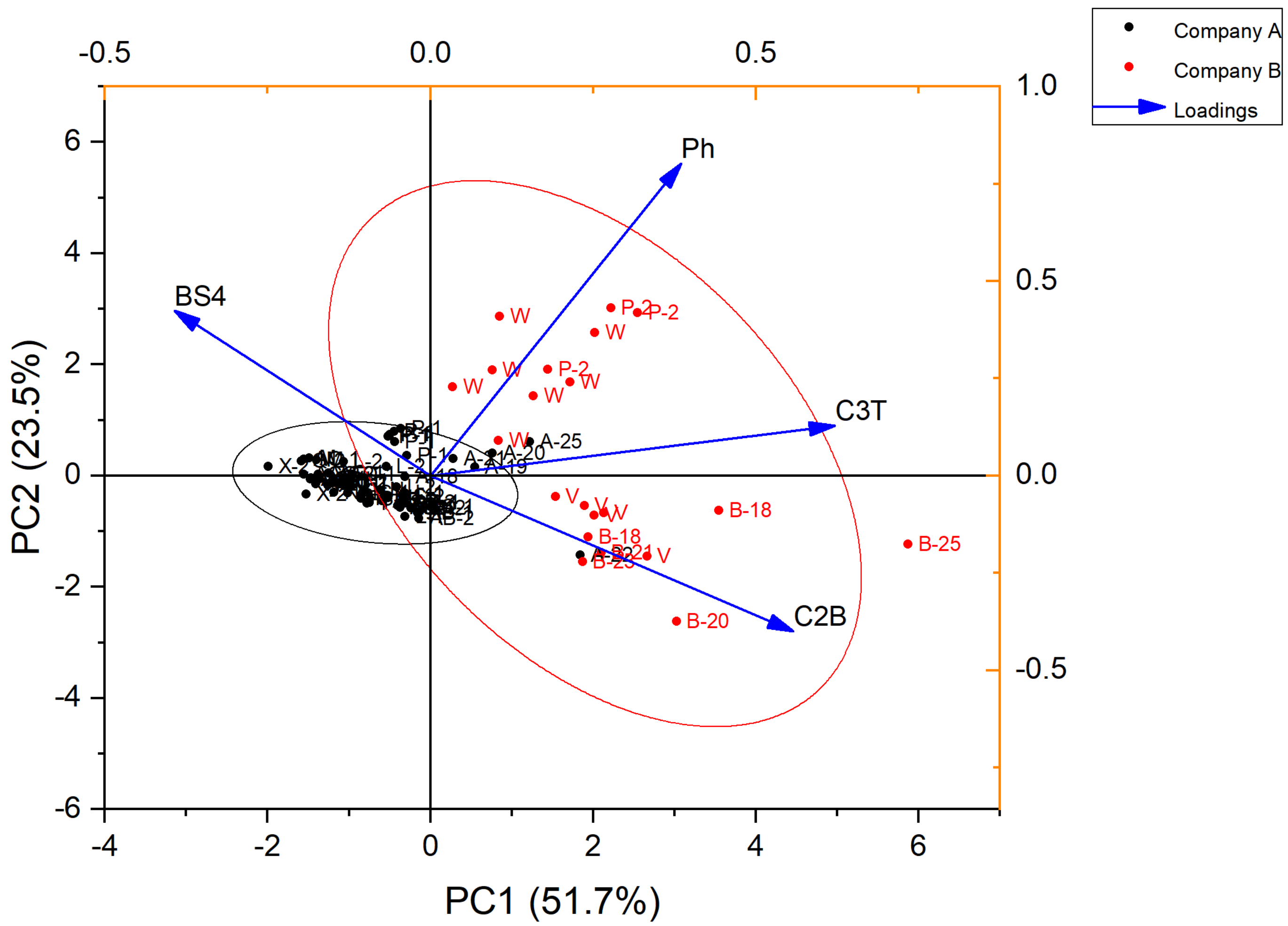

Figure S4. Baseline LDA manufacturing-era classification model using the conventional HDS $DR_{X/BS3}$ variable. The baseline LDA model uses $DR_{CxD/BS3}$, $DR_{C2B/BS3}$, $DR_{C3N/BS3}$, and $DR_{X/BS3}$ as predictor variables, with X set to C_3T. This model is provided as the baseline reference for comparison with the subsequent LDA models in which the C_3T DR is substituted with C_0De or C_1De DRs [11].

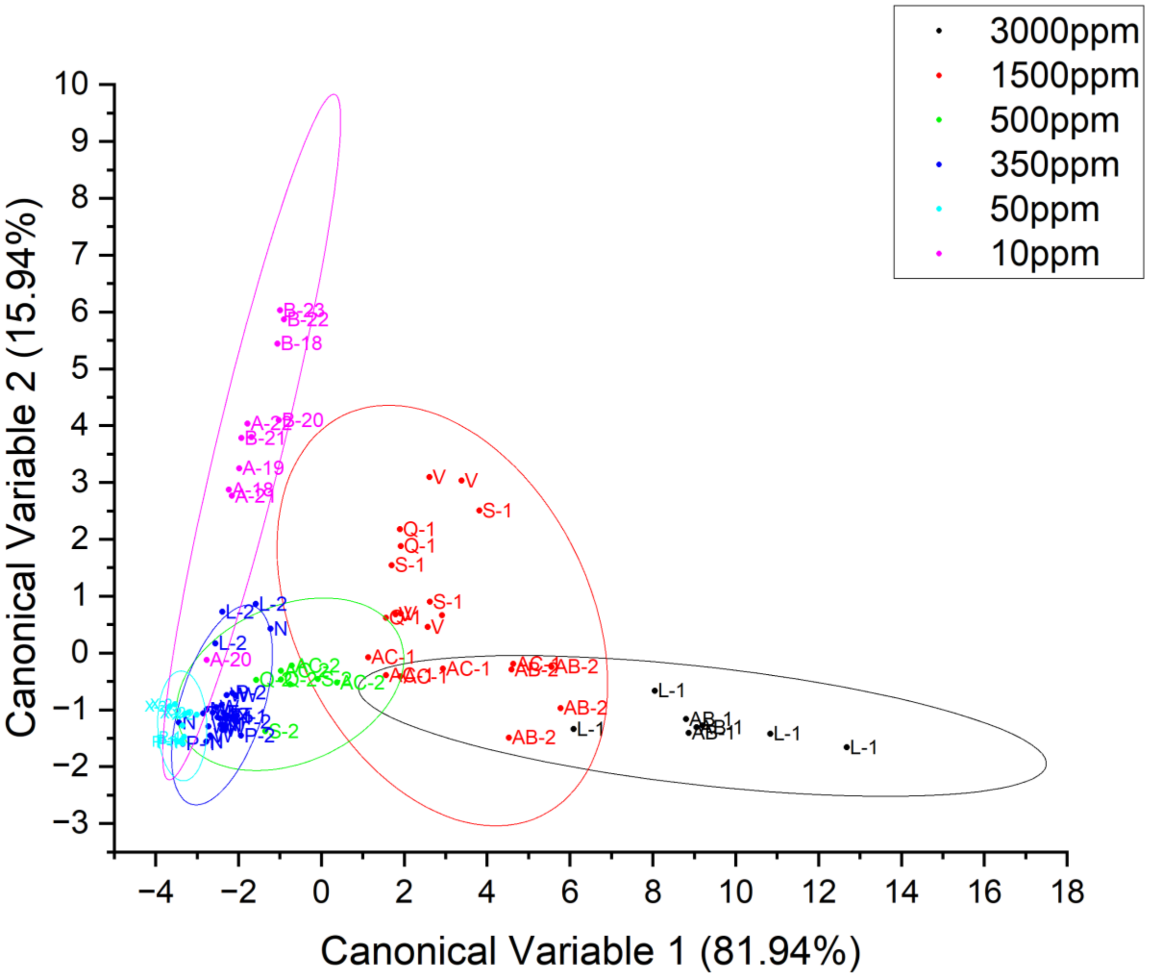

Figure S5. Representative extracted ion chromatograms (EICs) of C_0De and C_1De generated from full-scan GC/MS data. DR calculations for C_0De and C_1De in source identification and manufacturing-era estimation were based on the EIC of *m*/*z* =95. The additional superimposed EICs at *m*/*z* 95, 138, and 152 were included as confirmatory traces for the molecular ion masses of C_0De (*m*/*z* 138) and C_1De (*m*/*z* 152), respectively.

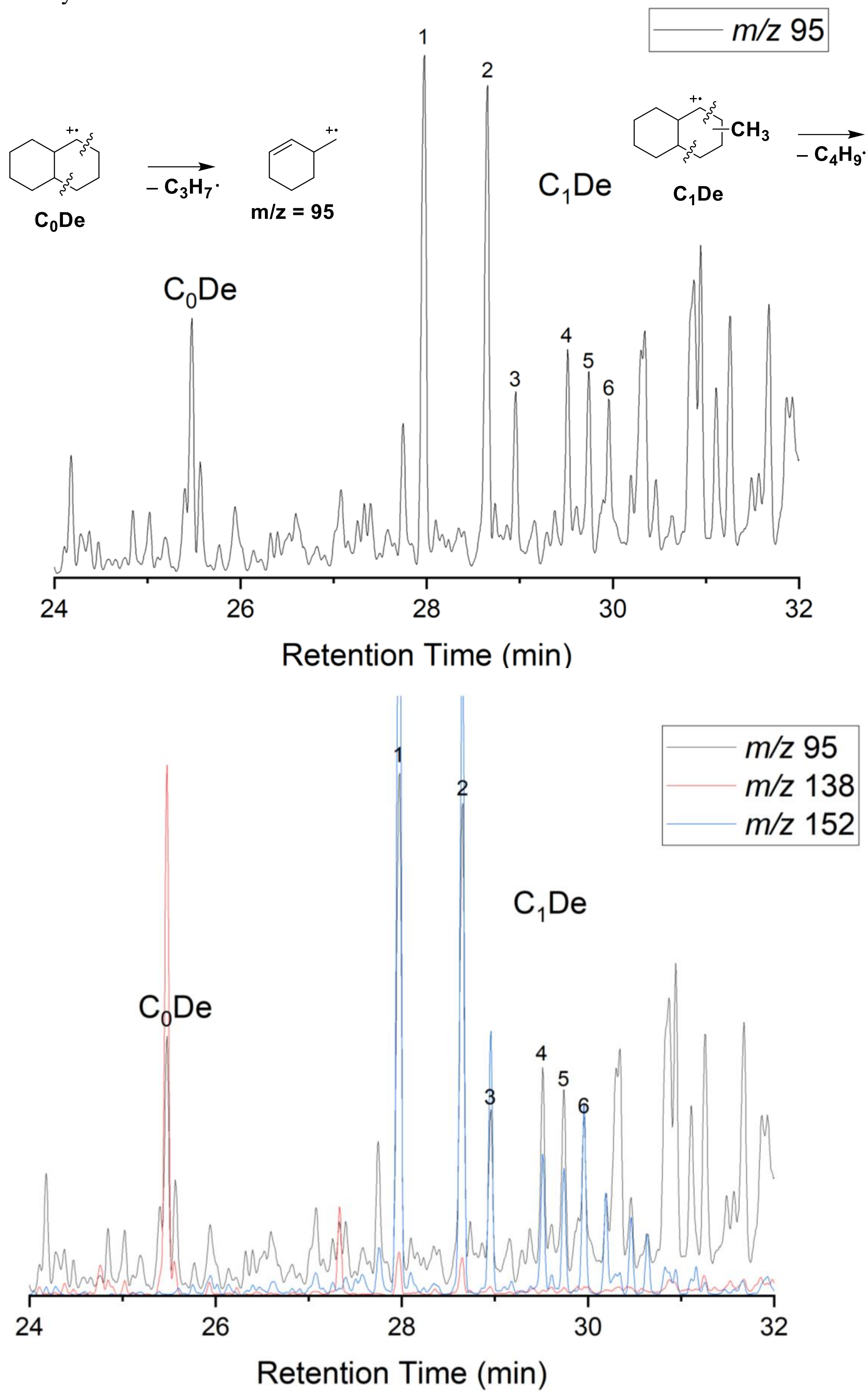

Supplement: Supplementary file 1 [file molecules-31-03006-s001.zip › molecules-4462069-supplementary.pdf]
